# Assessment of the Activity of Nitroisoxazole Derivatives against *Trypanosoma cruzi*

**DOI:** 10.3390/molecules29122762

**Published:** 2024-06-11

**Authors:** Mauricio Moncada-Basualto, Jorge Saavedra-Olavarría, Paula S. Rivero-Jerez, Cristian Rojas, Juan D. Maya, Ana Liempi, Matías Zúñiga-Bustos, Claudio Olea-Azar, Michel Lapier, Edwin G. Pérez, Josué Pozo-Martínez

**Affiliations:** 1 Instituto Universitario de Investigación y Desarrollo Tecnológico, Universidad Tecnológica Metropolitana, San Joaquín 8940577, Chile; mmoncadab@utem.cl (M.M.-B.); cristianrojas@udla.edu.co (C.R.); mzunigab@utem.cl (M.Z.-B.); 2 Department of Organic Chemistry, Faculty of Chemistry and Pharmacy, Pontificia Universidad Católica de Chile, Av. Vicuña Mackenna 4860, San Joaquin 7820436, Chile; jorge.saavedra@uc.cl (J.S.-O.); psrivero@uc.cl (P.S.R.-J.); 3 Laboratory of Free Radicals and Antioxidants, Faculty of Chemical and Pharmaceutical Science, Universidad de Chile, Olivos 1007, Independencia 8380544, Chile; colea@uchile.cl; 4 Programa de Farmacología Molecular y Clínica, Instituto de Ciencias Biomédicas, Facultad de Medicina, Universidad de Chile, Av. Independencia 1027, Independencia 8380453, Chile; jdmaya@uchile.cl; 5 Programa de Biología Integrativa, Instituto de Ciencias Biomédicas, Facultad de Medicina, Universidad de Chile, Av. Independencia 1027, Independencia 8380453, Chile; anitavet@gmail.com; 6 Centro de Investigación, Desarrollo e Innovación de Productos Bioactivos (CinBio), Escuela de Química y Farmacia, Facultad de Farmacia, Universidad de Valparaíso, Av. Gran Bretaña 1093, Valparaiso 2360102, Chile; michel.lapier@uv.cl; 7 Laboratorio de Química—Médica, Facultad de Ciencia y Tecnología, Universidad del Azuay, Av. 24 de Mayo 777, Cuenca 010204, Ecuador

**Keywords:** Chagas disease, *Trypanosoma cruzi*, nitroisoxazoles, ROS, cruzipain enzyme

## Abstract

The development of new compounds to treat Chagas disease is imperative due to the adverse effects of current drugs and their low efficacy in the chronic phase. This study aims to investigate nitroisoxazole derivatives that produce oxidative stress while modifying the compounds’ lipophilicity, affecting their ability to fight trypanosomes. The results indicate that these compounds are more effective against the epimastigote form of *T. cruzi*, with a 52 ± 4% trypanocidal effect for compound **9**. However, they are less effective against the trypomastigote form, with a 15 ± 3% trypanocidal effect. Additionally, compound **11** interacts with a higher number of amino acid residues within the active site of the enzyme cruzipain. Furthermore, it was also found that the presence of a nitro group allows for the generation of free radicals; likewise, the large size of the compound enables increased interaction with aminoacidic residues in the active site of cruzipain, contributing to trypanocidal activity. This activity depends on the size and lipophilicity of the compounds. The study recommends exploring new compounds based on the nitroisoxazole skeleton, with larger substituents and lipophilicity to enhance their trypanocidal activity.

## 1. Introduction

American Trypanosomiasis, also known as Chagas disease (CD), is a neglected parasitic disease caused by the *Trypanosoma cruzi* (*T. cruzi*) parasite. It is mainly transmitted by blood-sucking insects of the Triatominae subfamily [1,2]. The World Health Organization estimates that it affects around 7 million people worldwide, causing approximately 10,000 deaths per year [3]. Although the risk of infection is related to poverty and inequality, globalization has also made the disease a problem in non-endemic countries [4,5]. The life cycle of the *T. cruzi* parasite includes three morphological forms: epimastigote, trypomastigote, and amastigote. It begins when the vector feeds on the blood of a mammal. Trypomastigotes differentiate into epimastigotes in the triatomine midgut and then into infective metacyclic trypomastigotes in the hindgut. The parasite can enter the host through the skin or mucosa, invade many nucleated cells, and replicate intracellularly. Infection of specific tissues persists indefinitely for the host’s life, indicating the chronic phase of the disease [6,7,8].

The CD presents two distinct clinical phases: an acute phase, which appears shortly after infection and is characterized by high parasitemia but is asymptomatic in most cases, and a chronic phase, which can last for many years and is characterized by low or no parasitemia. After years or decades without symptoms, the chronic phase can lead to serious health problems, such as chronic heart failure, digestive damage (megacolon and megaesophagus), and peripheral nerve damage. Currently, drug therapies for CD are limited to nifurtimox (Nfx) and benznidazole (Bzn), especially effective during the early acute phase, provided patients complete an entire 60-day course at the correct dose. However, in the chronic phase, the effectiveness of these drugs is diminished, and they can cause side effects. Therefore, investigating new therapies against *T. cruzi* is still necessary [7,8,9,10,11]. The mechanism of action of the drugs Nfx and Bzn against CD has yet to be fully understood. Still, it is known that both compounds require the reduction in their nitro groups by type I nitroreductases (NTRs) present in the parasite. This reduction leads to the formation of electrophilic metabolites or highly reactive radical species that can damage the intracellular macromolecules of the parasite and ultimately induce its death. The presence of the nitro group and its requirement for bioactivation by NTRs are believed to be critical to the trypanocidal activity and selectivity of the drugs [12,13].

Currently, research related to CD is focused on the rational design of compounds with improved trypanocidal activities, considering that these activities are selective for targets essential to the parasite’s survival without affecting the host [9]. In this regard, the study of derivatives of the isoxazole nucleus is interesting, as this aromatic heterocycle has demonstrated engaging trypanocidal activities as well as biological activities such as anti-Alzheimer’s, antiviral, anti-inflammatory, antidiabetic, anticonvulsant, antineoplastic, and antioxidant effects that support its pharmacological potential [14,15,16,17,18]. Da Rosa et al. [16] described the trypanocidal activity of a new series of 3,5-disubstituted isoxazoles, active against intracellular amastigotes of *T. cruzi* (Tulahuen), as well as the cytotoxicity of these compounds in THP-1 cells. They found that some of the obtained compounds are interesting starting scaffolds for the generation of potentially valuable compounds as therapies against CD, especially derivative 31-a, which showed good trypanocidal activity (IC_50_: 1.13 µM), low cytotoxicity (IC_50_: 182 µM), and good selectivity (SI: 161). Additionally, the authors identified that this compound could inhibit the enzyme trypanothione reductase, which is essential for *T. cruzi* metabolism. Similarly, de Souza et al. studied a new series of 3,5-diaryl isoxazoles for their anti-*T. cruzi* activity in vitro against the trypomastigote and amastigote forms of the parasite, as well as their cytotoxicity in L929 cells [18]. They found that these compounds possess trypanocidal activities, mainly analog 7b (IC_50_: 3.3 µM), with an SI of 26.4. Furthermore, Zimmermann et al. studied the trypanocidal activity against amastigotes form of *T. cruzi* and cytotoxicity in THP-1 (CC_50_) of a series of bis-heterocyclic isoxazole-triazole compounds [17]. They found that the analyzed compounds are also promising scaffolds for the development of therapies against *T. cruzi*, incredibly derivative 31-b, which showed good trypanocidal activity (IC_50_: 12.2 µM) and a selectivity index (SI: >41) like that of Bzn.

The enzyme cruzipain is an essential target for the parasite’s survival that is not found in mammals. It is a 57 kDa protein in all parasite forms. It is a necessary antigen for *T. cruzi*, stimulating the host with macrophages, enhancing arginase activity, and promoting the parasite’s propagation [19,20]. Studies carried out by Bellera et al. determined that the trypanocidal activity of the drug benipidine is due to the enzymatic inhibition of cruzipain, preventing the differentiation from trypomastigote to amastigote in vitro models [21]. Specifically, the nitrophenyl of benipidine interacts with the amino acid residues of the enzyme’s active site.

**Figure 1:**
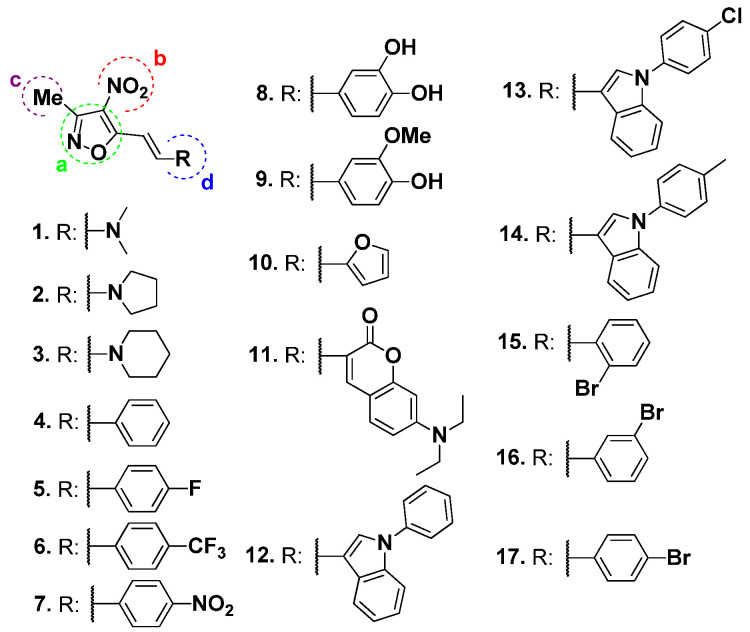
Serie of nitroisoxazoles derivatives in the study.

Based on the background presented in this work, the study proposes investigating a series of nitroisoxazole derivatives. Structurally, these compounds consist of (a) the isoxazole nucleus, which has been linked to trypanocidal activities associated with the inhibition of cruzipain, (b) the nitro function associated with trypanocidal activity linked to the generation of intracellular oxidative damage through activation by NTRs, (c) a methyl group that contributes to the lipophilicity of the compounds associated with their bioavailability, and (d) variations in length (Figure 1).

## 2. Results and Discussion

### 2.1. Synthesis of Derivates

**Scheme 1:**
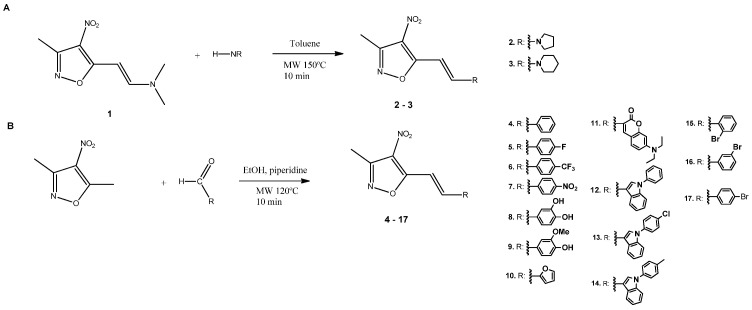
Synthetic methodology for obtaining compounds **1**–**17**. (**A**) Synthesis of derivates of nitroisoxazoles **2**–**3**. (**B**) Synthesis of nitroisoxazoles **4**–**17**.

The described derivatives were efficiently synthesized; compound 1 was synthesized according to the procedure described by Dere et al. [22]. Compounds **2** to **3** were obtained efficiently following the protocol outlined in Scheme 1. Derivatives **2** and **3** were obtained from compound **1** and the secondary amine. The reaction was carried out in a Monowave reactor at 150 °C for 10 min in toluene solvent. Under these conditions, the respective enamine was formed. For derivatives, **4** to **17** (Scheme 1), 3,5-dimethyl-4-nitroisoxazole and the respective aldehyde were added to a cell in ethanol, and piperidine was heated at 120 °C for 10 min in a Monowave reactor. The obtained derivatives were washed several times with ethanol to remove the presence of piperidine.

### 2.2. Electrochemical Studies

The chemistry mechanism of reduction in nitroisoxazole derivatives was investigated using cyclic voltammetry. The compounds exhibited an electrochemical behavior like that observed in other nitro compound families [23,24,25].

**Figure 2:**
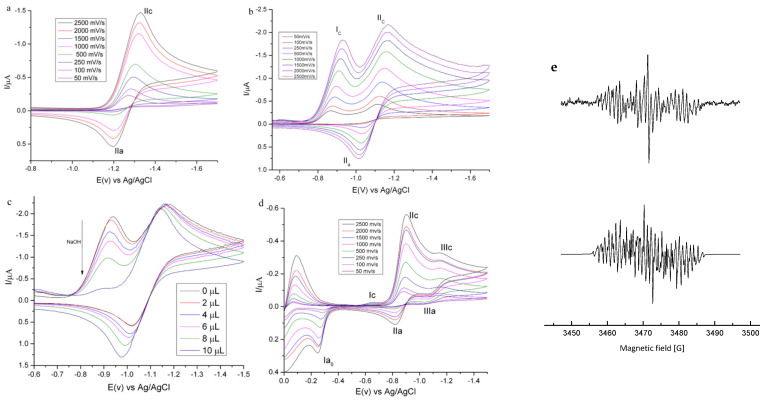
(**a**) Cyclic voltammogram of compound **2** with a potential sweep between −1.7 and −0.8 V and sweep speeds between 0.05 and 2.5 V/s. (**b**) Cyclic voltammogram of compound **8** with a potential sweep between −1.7 and −0.6 V and sweep speeds between 0.05 and 2.5 V/s contains labile protons. (**c**) Cyclic voltammogram of compound **8** using speed 2.0 V/s, (i) the first black line without NaOH; (ii) the purple line, in the presence of NaOH 1 M. (**d**) Cyclic voltammogram of compound **9** with a potential sweep between −1.5 and 0.0 V and sweep speeds between 0.05 and 2.5 V/s. (**e**) Experimental (upper) and WimSIM 9 (down) simulated the ESR spectrum of compound **7** at room temperature in DMSO.

Figure 2a shows the cyclic voltammogram of compound **2**, which exemplifies compounds without labile protons in their structure (except compound **8**). The peak near −1.32 V corresponds to a quasi-reversible one-electron transfer, according to the criteria of Nicholson–Shain [26] (Table 1), attributable to the species ${-NO}_{2}/{NO}_{2}^{.-}$ . The latter species stable anion radical at room temperature. Figure 2b shows the voltammogram of compound 8, which exhibits labile protons in its structure. An irreversible peak at −0.93 V to the mono-electronic transfer ${-HNO}_{2}/H{NO}_{2}^{.-}$ is observed, coupled with a chemical reaction associated with autorotation of the formed radical. This was evidenced by the addition of NaOH (1 M), which caused a decrease in the Ic peak (Figure 2c).

Figure 2d shows the voltammogram of compound **9**, where an IIIc couple is observed at −1.14 V, corresponding to the formation of a hydroxylamine derivative. As previously described, an irreversible signal assigned to hydroxylamine oxidation to a nitroso derivative is also observed for N-acylhydrazones organometallic compounds with a pendant 5-nitrofuryl group [23,27]. Nitroisoxazoles containing an amino group exhibit more negative reduction potentials than derivatives containing aromatic groups in their structure due to the electron-donating effect of the amino group (Table 1). Compound **7**, which has the least negative reduction potential, can be attributed to introducing a nitro group in the para position of the nitroisoxazole ring. This effect can also be observed in derivatives containing halogens in the same position.

The reduction potentials of nitroisoxazoles were similar to those of 5-nitroindazoles previously studied by the group [24,28,29]. For the latter, trypanocidal activity was associated with the generation of oxidative stress and a relationship with the reduction potentials of the nitro group. Some compounds in the series under study exhibit potentials similar to nifurtimox, whose mechanism of action involves its bio-reduction. Due to this, it is postulated that the explored series of nitroisoxazoles will be active against the infective form of *T. cruzi*.

The reduction potentials recorded for compounds containing aromatic groups in their structure were those described for 5-nitrofuran derivatives [24] and for organometallic compounds derived from 4- and 5-nitrothiophene [30].

### 2.3. Monitoring of Electrochemically Generated Radicals by ESR

To elucidate the reduction mechanism of this series of compounds, ESR spectra were recorded by performing in situ electrolytic generation of the free radicals using the potentials obtained from cyclic voltammetry for the IIc couple. (Table S1) shows the hyperfine coupling constants obtained from the semi-empirical simulation of the spectra. ESR spectra of compounds containing an amino group in their structure (Figure 2a) indicate the formation of a nitro radical with the unpaired electron delocalized in the double bond of the spacer connecting the amino group to the nitro isoxazole ring for compounds containing aromatic groups in their structure (Figure 2b) (Scheme S1).

### 2.4. Capacity Antioxidant by ORAC-FL

To corroborate the generation of the nitro radical, the antioxidant capacity of the series under study was determined by ORAC-FL methodology, employing a fluorescent probe to capture oxygen-centered radicals (Table 2). The ORAC-FL values of the studied compounds are close to the reference value of Trolox, indicating a low antioxidant capacity. For compounds (**1**–**3**), the presence of amino substituents decreases antioxidant activity due to their electron-attracting characteristics, preventing the presence of labile hydrogens. Compound 1 exhibited the highest activity among the three, possibly explaining that the electron pair of the nitrogen in the tertiary amine would be more susceptible to be donated to stabilize the radical, which is not observed in heterocycles [31]. Compounds **4** and **10** present similar ORAC indexes as aromatic rings with high electron density insert the transfer of labile protons [32].

Substituents in the aryl group, such as halogens and nitro, found in compounds **5**–**7** and **15**–**17**, have been shown to reduce antioxidant activity. This is due to the electron-attracting nature of these groups, which eliminates labile protons. Additionally, the presence of the nitro group in the nitro isoxazole section of the molecule has a similar effect, further reducing antioxidant capacity [33]. In compound **11**, the presence of an amino group in the coumarin decreases antioxidant capacity, consistent with findings by Todorov et al. [34], who determined that the presence of an amino group as a substituent in the coumarin base skeleton dramatically decreases antioxidant capacity.

For derivatives **12**–**14**, when aromatic rings substitute the nitrogen of the indole, antioxidant activity decreases as the electron pair in the hydrogen atom of the heterocycle is hindered, being the most reactive part of the indole [35]. Among the series, compound **8**, which has a catechol substituent in its structure, showed the highest antioxidant activity, similar to Trolox. Studies by Justino et al. [36] determined that the presence of a catechol group as a substituent increases antioxidant capacity, as the increased planarity of the molecule allows greater electron delocalization, providing stability to the generated radical.

This is corroborated by cyclic voltammetry, where compound **8** was found to have a labile hydrogen atom, which could be responsible for the antioxidant activity. For derivative **9**, where a methoxy group replaced a hydroxyl group of the catechol substituent, there is a decrease in antioxidant capacity caused by electron-attracting groups. In conclusion, the lack of labile hydrogen atoms in the structure of nitroisoxazole derivatives could confirm the generation of free radicals, supporting the proposal by Aravena et al. [37].

### 2.5. Biological Studies

#### 2.5.1. Trypanocidal and Cytotoxicity Activity

The determination of trypanocidal activity was conducted using the tetrazolium salt reduction methodology (3-(4,5-dimethylthiazol-2-yl)-2,5-diphenyltetrazolium bromide) (MTT). As a preliminary step, a study of nitroisoxazole derivatives on the epimastigote form of *T. cruzi* was performed. Despite the epimastigote form not being a clinical form present in mammals, its easy handling has allowed screening for the trypanocidal activity of various compounds. It is worth noting that, during the intracellular life cycle of the parasite, a form similar to the epimastigote, which is biochemically and morphologically identical to the form present in the vector, has been described [38]. Table 3 shows the percentage of toxic effects of nitroisoxazole derivatives and precursors of the compounds under study. Compounds **4**–**10**, **14**, **15** and **17** exhibited the highest trypanocidal effects in the series under study, with compound **9** showing the highest activity, although it did not surpass the drug Nfx. These results indicated that halogens or methoxy groups as substituents in nitroisoxazole derivatives modulated trypanocidal activity. This is consistent with studies by Fonseca-Berzal et al. [38], who determined that 5-nitroindazole derivatives with halogen substituents showed the best activity against epimastigotes, as the electron-attracting characteristics of these substituents modify the physicochemical properties of molecules to improve their pharmacological properties.

Additionally, compounds with higher activity have aromatic rings as substituents for nitroisoxazoles, confirming what was described Do Vale Chaves e Mello et al. [39], who indicated that the presence of aromatic rings as substituents for nitroimidazole derivatives increases trypanocidal activity. Furthermore, a catechol group (**8**) would enhance trypanocidal activity, as observed with derivatives of 3-arylcoumarins studied by Pozo-Martínez et al. [40]. Moreover, the presence of a nitro group in the isoxazole seems to be crucial for trypanocidal activity, consistent with Toro et al. [23], who indicated that the presence of a nitro group as a substituent in a heterocyclic ring is more susceptible to reduction, leading to the formation of trypanocidal products. It has also been determined that the presence of an isoxazole is essential for activity, as its aromatic structure contains a weak nitrogen-oxygen bond that, under different reaction conditions, can undergo ring cleavage, increasing activity [41]. The results obtained on epimastigotes cannot be conclusive for determining potential Chagas agents, so conducting studies on the infective, extracellular, non-replicative trypomastigote form of *T. cruzi* present in humans is essential. Table 3 shows the results of the series under study, where a decrease in activity is observed compared to that obtained in the epimastigote form, except for compounds **3**, **11**, **12**, **13** and **16** which increased activity compared to the epimastigote form but were lower than the drug Nfx.

Da Rosa et al. [16], De Souza et al. [18] and Zimmermann et al. [17], determined that the most active isoxazole derivatives against *T. cruzi* trypomastigotes exhibited high lipophilicity.

Furthermore, Manso Alves et al. [42] determined differences in the glycoprotein composition of the cell membranes of trypomastigotes and epimastigotes. They identified 170 and 334 units of glycoproteins for *T. cruzi* epimastigotes and trypomastigotes, respectively. This finding suggests that the cell membrane of trypomastigotes is more lipophilic than that of epimastigotes, supporting the obtained results, as the compounds that were more active against the trypomastigote form have high LogP values, while against the epimastigote form, they have moderate LogP values.

IC_50_ values were determined for the most active compounds against both parasitic forms. Additionally, the cytotoxicity of the compounds on VERO mammalian cells was assessed (Table 4). The IC_50_ values obtained for the most active compounds were 400 µM or higher for the trypomastigote form, while for the epimastigote form, only compound **9** exhibited a lower IC_50_: 166 ± 4 µM. It is observed that the majority of the derivatives present considerable cytotoxicity.

Based on the results, two postulations are made: (1) lipophilicity is likely the primary modulator of trypanocidal activity in isoxazole rings, and (2) introducing the nitro group is important to the activity of the compounds. De Souza et al. [18] partially support this latter hypothesis for derivatives of 3,5-diarylisoxazole. Therefore, it would be essential to evaluate a possible mechanism of action of nitroisoxazole derivatives by generating oxidative stress in the parasite.

#### 2.5.2. Generation of ROS on *T. cruzi*

To generate radical species on *T. cruzi* trypomastigotes, intracellular free radicals were determined using the fluorescent probe dichlorofluorescein (DCF). For this purpose, the nitroisoxazole derivatives that showed the best trypanocidal activity were used.

**Figure 3:**
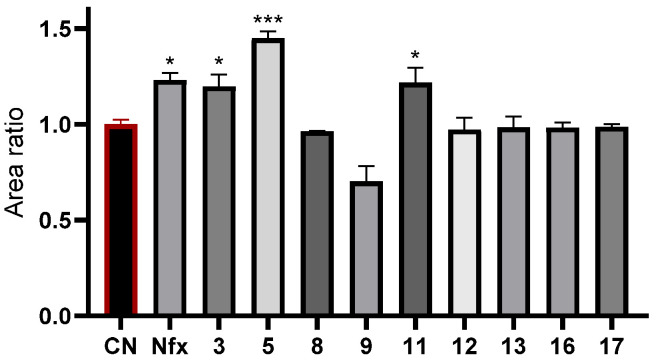
Ratio area of the ROS generation of the series of nitroisoxazoles on *T. cruzi*. The significant difference compared to the control **CN** (trypomastigotes without compound) (one-way ANOVA with Dunnett’s post-test, * *p* < 0.05, *** *p* < 0.001 statistical difference signific with **CN**).

Compounds **3**, **5**, and **11** showed an increase in fluorescence intensity, indicating the generation of free radicals (Figure 3). Compound **5** generated more reactive oxygen species (ROS) than Nfx, while compounds **3** and **11** exhibited similar values to the drug.

Furthermore, this experiment indicated that the compounds could cross the parasite’s membrane, providing information on membrane permeability models in vitro [43].

This assay identified a possible mechanism of action for the most active nitroisoxazole derivatives, which involves the generation of radical species. This confirms that a nitro group in the base skeleton of the isoxazole heterocycle modulates trypanocidal activity. Compounds **8**, **9**, **12**, **13**, **16**, and **17**, which did not show ROS generation, may have a different mechanism of action. Studies carried out by Bellera et al. [21], determined that the drug benipidine, which contains a nitro group attached to an aryl, exhibits inhibitory activity against the enzyme cruzipain. Similarly, Diaz-Urrutia et al. [44], studied a series of indoles, where a nitro group attached to the indazole generated inhibition of the enzyme trypanothione reductase. Therefore, given the low activity of the compounds under study, despite the generation of intracellular ROS similar to the Nfx drug, it suggests targets associated with enzyme inhibition.

Based on the above, an alternative was explored to explain the activity of nitroisoxazole derivatives against *T. cruzi* due to the structural similarity of these compounds with thiazole derivatives [45], which are inhibitors of the enzyme cruzipain, a cysteine lysosomal protease. During the infection process, the activation of this cysteine protease is essential for the parasite since it allows the penetration of the trypomastigote into mammalian cells and its differentiation into other cell forms to continue the life cycle [46,47,48,49,50]. Which makes cruzipain an exciting target for study. Therefore, a docking analysis of the nitroisoxazole derivatives on the enzyme was performed in this study, as this is the first step to identify potentially active compounds.

#### 2.5.3. Inhibition of Cruzipain Enzyme on *T. cruzi*

**Figure 4:**
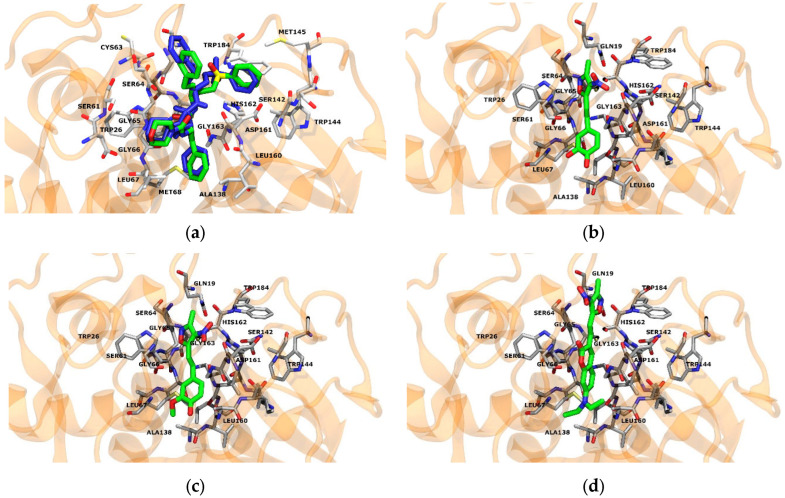
(**a**) Superposition between native crystallographic ligand and native docked structure, (**b**) Binding modes of compound **8**, (**c**) **9**, and (**d**) **11** through docking.

Docking simulations were carried out as described by Caputto et al. [51]. The structure of cruzipain was obtained from Protein Data Bank ID: 1F29. AutoDockGPU was used to calculate optimal energy conformations for ligands that interact with the active site of the protein, including the active 3-[[N-[morpholin-N-yl-carbonyl]-phenylalaninyl-amino]-5-phenyl-pentane-1-sulfonylbenzene ligand as docking control (native). Table 5 shows the docking score values obtained for each compound. In Figure 4b–d, it can be observed the best three compounds (**8**, **9** and **11**) enter the active site pocket of cruzipain, generating interactions with some of the relevant amino acid residues utilized by the native compound.

In detail, compound **8** displayed a hydrogen bond with G66, M68 and E205 residues, and hydrophobic interactions with L67 and A133. Similarly, compound **9** exhibits hydrogen bonds with G66, M68 and E205 residues and hydrophobic contacts with L67, A133 and L157.

In Figure 4b–d, it can be observed the best three compounds (**8**, **9** and **11**) enter the active site pocket of cruzipain, generating interactions with some of the relevant amino acid residues utilized by the native compound. In detail, compound **8** displayed a hydrogen bond with G66, M68 and E205 residues, and hydrophobic interactions with L67 and A133. Similarly, compound **9** exhibits hydrogen bonds with G66, M68 and E205 residues and hydrophobic contacts with L67, A133 and L157.

Compound **11** revealed hydrogen bonds with W26 and G66, and hydrophobic contacts with L67, L157 and E205. The possible reasons for the low interaction energy values compared to the control could be the differences in the hydrogen bond and hydrophobic interactions and the size of the nitroisoxazole compounds, as they are smaller when compared to the native compound, which could lead to instability of the compounds in the active site. The compounds only shared the G66 hydrogen bond and the hydrophobic contact with L67, with native ligand.

From the descriptors of all the compounds in this series, we obtained the radars of physicochemical properties that allow us to determine the optimal structures for the design of new drugs; among these, we can highlight the unsaturations descriptor, which in the different structures of the series is increased, this parameter indicates that the molecule has an excess of double bonds that could interfere with the biological activity of the molecules. Furthermore, descriptors such as polar surface area (TPSA), size, lipophilicity, flexibility, and solubility meet the optimal ranges for each property (Supplementary Materials). On the other hand, all the descriptors related to bioavailability, Lipinski’s Rule of Five criteria, drug-likeness, and ADME parameters meet the criteria (Table S2), especially compound **9**, which presents interesting trypanocidal activity, which meets the ADME criteria. However, when looking at the cytochrome descriptors (CYP), these are enzyme inhibitors. Derivatives of all compounds have a high potential to be structurally improved and generate new molecules with trypanocidal activity.

Based on the information provided, future studies should focus on designing a new series of compounds that retain the nitroisoxazole skeleton as a base for generating free radicals.

Furthermore, it would be interesting to explore the incorporation of different substituents in the heterocycle to modulate the lipophilicity of these compounds. For example, the introduction of large straight or branched chains, coumarins, quinolones, and other similar groups could be considered. These modifications can improve the interaction energy of the compounds with the cruzipain enzyme, which leads to the evaluation of other possible mechanisms of action of these compounds. In addition, a dual effect of the compounds could be generated when evaluating the inhibition of the enzyme trypanothione reductase, responsible for the antioxidant protection of the parasite.

One such pathway might involve testing for its ability to inhibit cellular respiration, as this could disrupt essential metabolic processes in the parasite. Furthermore, it might be of value to explore the possible effects of depolarization on the parasite’s mitochondrial membrane, as it may disrupt energy production and parasite viability.

## 3. Materials and Methods

### 3.1. Synthesis

All solvents, including deuterated solvents, were purchased from Merck. Other reagents were from Aldrich (Saint Louis, MO, USA), Merck (Boston, MA, USA), or AK Scientific (Ahern Ave, CA, USA). Melting points were determined on a Reichert Galen III hot plate microscope apparatus and were uncorrected. NMR spectra were recorded on a Bruker Avance 400 MHz spectrometer (Billerica, MA, USA). All chemical shifts in NMR experiments are reported as ppm downfield from TMS. The following calibrations were used: CDCl3 δ = 7.26 and 77.0 ppm for ^1^H NMR and ^13^C NMR, respectively. C, H, N and S analyses were carried out with a Thermo Scientific Flash (Waltham, MA, USA) 2000 elemental analyzer.

#### 3.1.1. Synthesis of (*E*)-*N*,*N*-Dimethyl-2-(3-methyl-4-nitroisoxazol-5-yl)ethen-1-amine (**1**) 

Compound **1** was synthesized according to the reported literature procedure, and the product was obtained as a golden yellow solid, mp: 154–156 °C. ^1^H NMR (400 MHz, DMSO) δ 8.00 (d, *J* = 13.0 Hz, 1H), 5.75 (d, *J* = 13.0 Hz, 1H), 3.25 (s, 3H), 2.98 (s, 3H), 2.39 (s, 3H). ^13^C NMR (101 MHz, DMSO) δ 170.04, 155.48, 153.89, 120.88, 80.71, 45.46, 37.56, 12.31 [22].

##### General Procedure to Obtain Compounds **2** and **3**

A G10 reaction vial was charged with **1** (394 mg, 2.0 mmol), toluene (4 mL), and the respective secondary amine (10.0 mmol). The reaction vial was capped, placed in the Monowave Reactor (Graz, Austria), and heated at 150 °C for 10 min. Once the reaction was deemed complete, through verification using TLC analysis, the toluene was evaporated under reduced pressure. This process yields **2** and **3** without the need for further purification.

#### 3.1.2. (*E*)-3-Methyl-4-nitro-5-(2-(pyrrolidin-1-yl)vinyl)isoxazole (**2**) 

Pale orange solid (99% yield), mp: 145–147 °C. ^1^H NMR (400 MHz, CDCl_3_) δ 7.85 (d, *J* = 13.1 Hz, 1H), 5.81 (d, *J* = 13.1 Hz, 1H), 3.58 (t, *J* = 6.8 Hz, 2H), 3.34 (t, *J* = 7.0 Hz, 2H), 2.47 (s, 3H), 2.06 (q, *J* = 6.8 Hz, 2H), 1.98 (q, *J* = 6.7 Hz, 2H). ^13^C NMR (101 MHz, CDCl_3_) δ 169.99, 155.81, 147.76, 82.89, 52.90, 47.21, 25.24, 25.06, 12.22 [22].

#### 3.1.3. (*E*)-3-Methyl-4-nitro-5-(2-(piperidin-1-yl)vinyl)isoxazole (**3**) 

Greenish yellow solid (30% yield), mp: 151–153 °C. ^1^H NMR (400 MHz, CDCl_3_) δ 7.54 (d, *J* = 13.3 Hz, 1H), 5.89 (d, *J* = 13.3 Hz, 1H), 3.38–3.32 (m, 4H), 2.38 (s, 3H), 1.68–1.56 (m, 6H). ^13^C NMR (101 MHz, CDCl_3_) δ 170.49, 155.61, 150.62, 121.76, 81.17, 55.55, 46.02, 26.49, 24.89, 23.80, 12.18 [22].

##### General Procedure to Obtain Compounds **4**–**17**

A G10 reaction vial was charged with 3,5-dimethyl-4-nitroisoxazole (284 mg, 2.0 mmol), ethanol (4 mL), and the respective aldehyde (2.0 mmol). The solution was stirred to homogenize, and piperidine (20 µL, 0.2 mmol) was added. The reaction vial was capped, placed in the Monowave Reactor (Anton Paar Monowave 50), and heated at 120 °C for 10 min. After cooling the reaction mixture to room temperature, the precipitate was filtered and washed several times with ethanol to give the compounds **4**–**17**.

#### 3.1.4. (*E*)-3-Methyl-4-nitro-5-styrylisoxazole (**4**) 

White solid (65% yield), mp: 176–178 °C. 1H NMR (400 MHz, CDCl_3_) δ 7.57 (d, *J* = 16.5 Hz, 1H), 7.50–7.39 (m, 3H), 7.29–7.19 (m, 3H), 2.38 (s, 3H). 13C NMR (101 MHz, CDCl_3_) δ 167.16, 156.14, 143.07, 134.42, 131.12, 129.17, 128.42, 110.95, 11.85 [52].

#### 3.1.5. (*E*)-5-(4-Fluorostyryl)-3-methyl-4-nitroisoxazole (**5**) 

Orange solid (55% yield), mp: 169–171 °C. ^1^H NMR (400 MHz, CDCl_3_) δ 7.84–7.49 (m, 4H), 7.14 (t, *J* = 8.6 Hz, 2H), 2.60 (s, 3H). ^13^C NMR (101 MHz, CDCl_3_) δ 166.97, 161.84, 156.14, 130.72 (d, *J* = 3.4 Hz), 130.36 (d, *J* = 8.6 Hz), 116.40 (d, *J* = 22.1 Hz), 110.69 (d, *J* = 2.6 Hz), 11.80. ^19^F NMR (376 MHz, CDCl_3_) δ −107.75 [52].

#### 3.1.6. (*E*)-3-Methyl-4-nitro-5-(4-(trifluoromethyl)styryl)isoxazole (**6**)

White solid (14% yield), mp: 187–189 °C. ^1^H NMR (400 MHz, DMSO) δ 8.06 (d, *J* = 8.1 Hz, 2H), 8.00 (d, *J* = 16.6 Hz, 1H), 7.85 (d, *J* = 8.2 Hz, 2H), 7.75 (d, *J* = 16.6 Hz, 1H), 2.53 (s, 3H). ^13^C NMR (101 MHz, DMSO) δ 166.39, 156.79, 140.91, 138.67, 130.79 (d, *J* = 31.8 Hz), 129.58, 129.25, 126.44 (q, *J* = 3.9 Hz), 125.80, 123.09, 113.82, 11.86. ^19^F NMR (376 MHz, DMSO) δ −61.31 [53].

#### 3.1.7. (*E*)-3-Methyl-4-nitro-5-(4-nitrostyryl)isoxazole (**7**) 

Yellow solid (15% yield), mp: 225–227 °C. ^1^H NMR (400 MHz, DMSO) δ 8.31 (d, *J* = 8.5 Hz, 2H), 8.11 (d, *J* = 8.8 Hz, 2H), 8.04 (d, *J* = 16.6 Hz, 1H), 7.79 (d, *J* = 16.6 Hz, 1H), 2.53 (s, 3H). ^13^C NMR (101 MHz, DMSO) δ 166.14, 156.86, 148.71, 141.00, 140.08, 130.03, 124.67, 115.08, 11.85 [53].

#### 3.1.8. (*E*)-4-(2-(3-Methyl-4-nitroisoxazol-5-yl)vinyl)benzene-1,2-diol (**8**)

Dark orange solid (57% yield), mp: 270 °C (decomposes). ^1^H NMR (400 MHz, DMSO) δ 7.75 (d, *J* = 16.4 Hz, 1H), 7.33 (d, *J* = 16.4 Hz, 1H), 7.21 (d, *J* = 2.1 Hz, 1H), 7.13 (dd, *J* = 8.3, 2.1 Hz, 1H), 6.83 (d, *J* = 8.2 Hz, 1H), 2.49 (s, 3H). ^13^C NMR (101 MHz, DMSO) δ 167.58, 156.49, 150.12, 146.40, 143.96, 127.43, 126.35, 123.23, 116.51, 114.63, 107.17, 11.98.

#### 3.1.9. (*E*)-2-Methoxy-4-(2-(3-methyl-4-nitroisoxazol-5-yl)vinyl)phenol (**9**) 

Pale orange solid (71% yield), mp: 189–191 °C. ^1^H NMR (400 MHz, DMSO) δ 9.89 (s, 1H), 7.81 (d, *J* = 16.4 Hz, 1H), 7.44 (d, *J* = 16.4 Hz, 1H), 7.39 (d, *J* = 2.0 Hz, 1H), 7.29 (dd, *J* = 8.2, 2.0 Hz, 1H), 6.88 (d, J = 8.2 Hz, 1H), 3.86 (s, 3H), 2.49 (s, 3H). ^13^C NMR (101 MHz, DMSO) δ 167.64, 156.53, 150.88, 148.56, 144.00, 127.48, 126.38, 124.14, 116.38, 112.21, 107.61, 56.23, 11.96 [54].

#### 3.1.10. (*E*)-5-(2-(Furan-2-yl)vinyl)-3-methyl-4-nitroisoxazole (**10**) 

Brown solid (63% yield), mp: 145–147 °C. ^1^H NMR (400 MHz, CDCl_3_) δ 7.55 (d, *J* = 1.8 Hz, 1H), 7.47 (s, 2H), 6.73 (dd, *J* = 3.6, 0.7 Hz, 1H), 6.51 (dd, *J* = 3.5, 1.8 Hz, 1H), 2.54 (s, 3H). ^13^C NMR (101 MHz, CDCl_3_) δ 167.07, 156.16, 151.10, 145.94, 128.63, 116.73, 113.01, 108.53, 11.86 [52].

#### 3.1.11. (*E*)-7-(Diethylamino)-3-(2-(3-methyl-4-nitroisoxazol-5-yl)vinyl)-2H-chromen-2-one (**11**)

Dark purple solid (47% yield), mp: 224–226 °C. ^1^H NMR (400 MHz, CDCl_3_) δ 8.02 (d, *J* = 16.3 Hz, 1H), 7.82 (s, 1H), 7.67 (dd, *J* = 16.3, 0.6 Hz, 1H), 7.32 (d, *J* = 8.9 Hz, 1H), 6.60 (dd, *J* = 8.9, 2.4 Hz, 1H), 6.47 (d, *J* = 2.5 Hz, 1H), 3.43 (q, *J* = 7.1 Hz, 4H), 2.55 (s, 3H), 1.22 (t, *J* = 7.1 Hz, 6H). ^13^C NMR (101 MHz, CDCl_3_) δ 167.66, 160.15, 156.97, 156.13, 152.27, 144.15, 137.95, 130.34, 114.49, 111.24, 109.80, 108.92, 97.09, 45.15, 12.51, 11.94.

#### 3.1.12. (*E*)-3-Methyl-4-nitro-5-(2-(1-phenyl-1H-indol-3-yl)vinyl)isoxazole (**12**)

Orange solid (77% yield), mp: 196–199 °C. ^1^H NMR (400 MHz, DMSO) δ 8.45 (s, 1H), 8.12 (d, *J* = 16.4 Hz, 1H), 8.05–7.98 (m, 1H), 7.75–7.55 (m, 6H), 7.56–7.48 (m, 1H), 7.43–7.32 (m, 2H), 2.48 (s, 3H). ^13^C NMR (101 MHz, DMSO) δ 168.10, 156.40, 138.15, 137.46, 137.12, 135.86, 130.46, 128.32, 124.93, 124.60, 123.21, 120.57, 114.69, 112.11, 106.34, 12.03. Anal. Calcd. for C_20_H_15_N_3_O_3_: C, 72.94; H, 4.59; N, 12.76. Found: C, 70.34; H, 4.67; N, 12.19.

#### 3.1.13. (*E*)-5-(2-(1-(4-Chlorophenyl)-1H-indol-3-yl)vinyl)-3-methyl-4-nitroisoxazole (**13**)

Orange solid (28% yield), mp: 217–220 °C. ^1^H NMR (400 MHz, DMSO) δ 8.42 (s, 1H), 8.09 (d, *J* = 16.5 Hz, 1H), 7.99 (dd, *J* = 6.8, 2.3 Hz, 1H), 7.69 (s, 4H), 7.61–7.53 (m, 2H), 7.43–7.29 (m, 2H), 2.47 (s, 3H). ^13^C NMR (101 MHz, DMSO) δ 167.99, 156.38, 137.28, 137.00, 136.84, 135.47, 132.53, 130.37, 126.61, 126.43, 124.69, 123.30, 120.56, 114.92, 112.01, 106.63, 12.01. Anal. Calcd. for C_20_H_14_ClN_3_O_3_: C, 63.25; H, 3.72; N, 11.06. Found: C, 63.10; H, 3.91; N, 10.86.

#### 3.1.14. (*E*)-3-Methyl-4-nitro-5-(2-(1-(p-tolyl)-1H-indol-3-yl)vinyl)isoxazole (**14**)

Reddish-orange solid (36% yield), mp: 208–211 °C. ^1^H NMR (400 MHz, DMSO) δ 8.25 (s, 1H), 8.07 (d, *J* = 16.4 Hz, 1H), 7.97 (d, *J* = 7.4 Hz, 1H), 7.55 (d, *J* = 16.4 Hz, 1H), 7.47 (t, *J* = 8.0 Hz, 3H), 7.41–7.25 (m, 4H), 2.46 (s, 3H), 2.39 (s, 3H). ^13^C NMR (101 MHz, DMSO) δ 168.26, 156.21, 137.94 (d, *J* = 3.2 Hz), 137.03, 135.85, 135.46, 130.78, 126.47, 124.83, 124.43, 123.00, 120.48, 114.66, 112.05, 106.49, 21.01, 11.71. Anal. Calcd. for C_21_H_17_N_3_O_3_: C, 70.18; H, 4.77; N, 11.69. Found: C, 70.37; H, 5.21; N, 11.61.

#### 3.1.15. (*E*)-5-(2-Bromostyryl)-3-methyl-4-nitroisoxazole (**15**)

Yellow solid (77% yield), mp: 169–172 °C. ^1^H NMR (400 MHz, DMSO) δ 8.06–7.96 (m, 2H), 7.78 (d, *J* = 8.0 Hz, 1H), 7.65 (d, *J* = 16.4 Hz, 1H), 7.53 (t, *J* = 7.6 Hz, 1H), 7.49–7.39 (m, 1H), 2.53 (s, 3H). ^13^C NMR (101 MHz, DMSO) δ 166.22, 156.85, 139.81, 134.00, 133.96, 133.04, 129.11, 128.87, 125.42, 114.27, 11.86. Anal. Calcd. for C_12_H_9_BrN_2_O_3_: C, 46.63; H, 2.93; N, 9.06. Found: C, 46.82; H, 3.24; N, 9.04.

#### 3.1.16. (*E*)-5-(3-Bromostyryl)-3-methyl-4-nitroisoxazole (**16**)

Yellow solid (90% yield), mp: 174–177 °C. ^1^H NMR (400 MHz, DMSO) δ 7.93 (s, 1H), 7.81–7.70 (m, 2H), 7.63–7.52 (m, 2H), 7.38 (t, *J* = 7.9 Hz, 1H), 2.48 (s, 3H). ^13^C NMR (101 MHz, DMSO) δ 166.62, 156.46, 141.09, 137.33, 133.78, 131.51, 131.36, 127.57, 122.91, 112.89, 11.52. Anal. Calcd. for C_12_H_9_BrN_2_O_3_: C, 46.63; H, 2.93; N, 9.06. Found: C, 46.94; H, 3.20; N, 9.07.

#### 3.1.17. (*E*)-5-(4-Bromostyryl)-3-methyl-4-nitroisoxazole (**17**)

Yellow solid (89% yield), mp: 195−198 °C. ^1^H NMR (400 MHz, DMSO) δ 7.90 (d, *J* = 16.6 Hz, 1H), 7.79 (d, *J* = 8.1 Hz, 2H), 7.69 (d, *J* = 7.7 Hz, 2H), 7.64 (d, *J* = 16.6 Hz, 1H), 2.50 (s, 3H). ^13^C NMR (101 MHz, DMSO) δ 166.74, 156.72, 141.64, 134.01, 132.64, 130.87, 125.00, 111.91, 11.89. Anal. Calcd. for C_12_H_9_BrN_2_O_3_: C, 46.63; H, 2.93; N, 9.06. Found: C, 46.84; H, 3.22; N, 9.04.

### 3.2. Electrochemical Studies

#### 3.2.1. Cyclic Voltammetry

Electrochemical characterization of the new compounds was performed through cyclic voltammetry (CV) on a 693 VA Metrohm instrument (Tokyo, Japan) equipped with a 694 VA Stand converter and 693 VA processor. Potentials were recorded against the Ag/AgCl reference electrode; a hanging drop of mercury (HDME) was used as a working electrode, and platinum was used as an auxiliary electrode. Tetrabutylammonium perchlorate (TBAP, 0.1 M) in DMSO was used as a supporting electrolyte under an N_2_ atmosphere at room temperature. Using Nicholson´s procedure [55], the Ipa/Ipc values (RNO_2_/RNO_2_^−^) were measured from each cyclic voltammogram with a scan rate between 0.1 and 2.5 V/s.

#### 3.2.2. Radical Generation by ESR-Electrochemical

ESR spectra were recorded in the X band (9.85 GHz) using a Bruker ECS106 spectrometer with a rectangular cavity and 50 kHz field modulation. The hyperfine splitting constants were estimated to be accurate within 0.05 G. The radicals were generated by an in situ electrolytic reduction process under the same experimental conditions as the CV in DMSO, with 0.1 M TBAP. The reduction potentials were acquired from CV. Every spectrum was obtained after 50 scans. ESR spectra were simulated using the program EPR-WinSIM Version 0.98 [56,57].

### 3.3. Oxygen Radical Antioxidant Capacity-Fluorescein (ORAC-FL)

Antioxidant capacity was determined using the ORAC-fluorescence methodology (ORAC-FL) [58]. The analysis used a 96-well white polystyrene plate reader (EnSpire multimode PerkinElmer, Waltham, MA, USA). The reaction was carried out in phosphate buffer (75 mM, pH 7.4). The compound solutions were prepared in dimethyl sulfoxide (DMSO, 0.8 to 10 µM), homogenized, and incubated at 37 °C, for 5 min, with 150 µL of fluorescein solution (40 nM, final concentration). Then, 25 µL of 2,2′-azobis(2-methylpropionamidine) dihydrochloride (Sigma–Aldrich 97%, St. Louis, MO, USA) 18 mmol·L^−^^1^ was added, and the fluorescence was registered (excitation wavelength of 485/20 nm and emission filter of 528/20 nm) at a constant temperature of 37 °C, in a Synergy HT multi-detection microplate reader (Bio-Tek Instruments, Winooski, VT, USA), every minute for 2 h. A blank with FL and AAPH, using dimethyl sulfoxide instead of the antioxidant solution, was used in each assay. Five calibration solutions using Trolox (0.1 to 10 µM) were also used in each assay. The inhibitor capacity was expressed as ORAC-FL values and quantified by integrating the area under the curve (AUCNET). All reaction mixtures were prepared in triplicate, and at least three independent assays were performed for each sample. The area under the fluorescence decay curve (AUC) was calculated by integrating the fluorescence decay where F_0_ is the initial fluorescence read at 0 min, and F is the fluorescence read at the time. The AUCNET corresponding to the sample was calculated by subtracting the AUC corresponding to the blank. Data were processed using Origin Pro 8.5 SR2 (San Francisco, CA, USA). The ORAC-FL indices were calculated according to the following Equation (1):

(1) $$ORAC-FLindex=\frac{\left({AUC}_{AH}-{AUC}_{CONTROL}\right)}{{(AUC}_{AH}-{AUC}_{CONTROL})}*\frac{\left[TROLOX\right]}{\left[AH\right]}$$

### 3.4. Biological Assays

#### 3.4.1. Trypanocidal and Cytotoxicity Activity

Epimastigotes (3 × 10^6^ parasites/mL) and trypomastigotes (10^7^ parasites/mL) or Vero cells (5 × 10^4^ cells) were incubated with the compounds re-suspend in DMSO in a culture medium (0.025% *v*/*v*, final concentration; epimastigote:LIT medium and, trypomastigote and Vero cell: RPMI 1640 medium) for 24 h. IC_50_ values were obtained by the dose–response analysis, where parasites or Vero cells were incubated with different concentrations (10, 50, 100, 200, 300, and 400 µM). The effects of the compounds on trypomastigote and Vero cell viability were evaluated by tetrazolium salt reduction assay (MTT). Briefly, 10 µL of 5 mgmL^−^^1^ MTT (3[4,5-dimethylthiazol-2-yl]-2,5-diphenyltetrazolium bromide) dye and 0.22 mgmL^−^^1^ phenazine methosulphate (used as an electron carrier) were added to each well containing 10^7^ parasites/mL (3 × 10^6^ for epismatigotes) or 5 × 10^4^ cells in 100 μL of RPMI 1640 medium, without phenol red. After incubating the parasites or cells for 4 h at 37 °C (28 °C for epimastigotes), the generated formazan crystals were dissolved on SDS 100 µL 10% (*w*/*v*) 0.01 M HCl. The plates were kept overnight at 37 °C (28 °C for epimastigotes), and the optical density (OD) was determined using a 96-well microplate reader (Vantaa, Finland) at 570 nm. Under these conditions, the OD is directly proportional to each well’s viable number of cells [40]. The experiments were performed in triplicate across three different assays.

#### 3.4.2. Generation of Reactive Oxygen Species (ROS) on Trypomastigotes of *T. cruzi*

For the detection of ROS, the 2′7′-dichlorodihydrofluorescein diacetate (DCFH_2_-DA) method was used 96-well plates were seeded with 10^7^ trypomastigotes/mL in RPMI 1640 medium without phenol red and supplemented with 5% SFBi (fetal bovine serum inactive). The cultures were incubated with a 20 µM DCFH_2_-DA solution for 15 min at 28 °C. Then, they were centrifuged at 3500 rpm and washed 2 times with phosphate buffer saline (PBS) pH 7.4. Cells loaded with DCFH_2_-DA were transferred to a Nunc^®^ fluorescence 96-well plate (Thermo Fisher Scientific Inc., Waltham, MA, USA), where the nitroisoxazoles derivates were added at 100 µM. Fluorescence (exi: 488 nm, emi: 528 nm) was recorded for 40 min in a BioTek (Winooski, VT, USA) Synergy HT spectrofluorometer. The area under increase curves over time was determined using Oring 8 software, with areas normalized to control. Results are the means ± SD of three independent experiments [43].

#### 3.4.3. Inhibition of Cruzipain Enzyme by Computational Docking Calculations and ADME Analysis

To understand the binding modes of the synthesized compounds, we performed molecular docking calculations, including the cruzipain enzyme as the receptor. The protein coordinates were retrieved from the Protein Data Bank (PDB ID: 1F29), and compounds (**1** to **11** and native) were prepared using Openbabel 3.1.0 software [59]. Autodock GPU v1.5 [60] package was employed for the docking calculations, setting a box size of 50 × 50 × 50 Å^3^ of size around the binding site with a grid space of 0.375 Å. The binding site was defined using the center of mass of the native compound 3-[[N-[morpholin-N-yl-carbonyl]-phenylalaninyl-amino]-5-phenyl-pentane-1-sulfonylbenzene reported in the crystallographic structure. Additionally, 1000 runs of the Lamarckian Genetic Algorithm and a Population size of 500 were employed in the protocol. The non-bonding interactions of the docked protein–compound complexes were estimated using the Protein–Ligand Interaction Profiler (PLIP) server [55].

A predictive computational theoretical model (Swiss ADME, http://www.swissadme.ch, accessed on 28 December 2023) of physicochemical properties, pharmacokinetics, drug similarity, and compatibility was used to evaluate the possible interactions of nitroisoxazole molecules with a biological target. With medicinal chemistry, including internal methods such as iLOGP (lipophilicity compounds predictor) and bioavailability radar (Pharmakocinetic parameters). Elements added to Lipinski’s 5 criteria rule provide essential information when synthesizing new molecules with biological interest. The compounds were entered through the computational SMILE nomenclature to generate the descriptors necessary for identifying each molecule [61].

### 3.5. Statistical Analysis

Statistical analysis was performed using Graph Pad Prism 4.03 (GraphPad Software, San Diego, CA, USA). The data are expressed as the mean SD of three independent experiments. Statistical analysis was performed using one–way ANOVA with Dunnett post-test. The date is considered statistically significant when *p* < 0.05.

## 4. Conclusions

In conclusion, the compounds studied have shown moderate to low trypanocidal activity, but promising evidence is that they generate oxidative stress in the parasite through the nitro radical anion. This is evidenced by cyclic voltammetry and the low antioxidant capacity evaluated by ORAC-FL. The compounds with better activity depend on lipophilicity, and the size of the compound is essential to increase activity in the case of enzymatic inhibition of cruzipain.

Based on these results, it is essential to continue investigating a series of compounds that maintain the basic skeleton of nitroisoxazole, known to modulate the generation of free radicals and induce oxidative stress in the parasite. Including different substituents that increase the compound’s size and lipophilicity, the trypanocidal activity is likely to be enhanced. It is also worth exploring other possible mechanisms of action, such as inhibiting various enzymes like cruzipain, arginine kinase, and *Tc*gapdh, among others.

This study is essential for developing new treatments for trypanosomiasis, a disease with limited therapeutic options. By continuing to investigate these compounds, we can find a more effective and safe treatment for this disease affecting millions worldwide.

## Figures and Tables

**Table 1:** Electrochemical parameters were obtained through cyclic voltammetry at 2.0 V/s for the series of nitroisoxazoles.

Compound	−Ep_cI_	−Ep_cII_	−Ep_aIII_	−Ep_aI_	−Ep_aII_	−Ep_aIII_	|ΔEp|	Ipa/Ipc
**1**		1.33			1.19		140	−0.44
**2**		1.32			1.20		120	0.32
**3**		1.29			1.19		100	−0.19
**4**		0.89	1.14		0.80	1.01	90	−0.09
**5**		0.82			0.71		110	−0.02
**6**		0.74	1.33					
**7**		0.71			0.64		70	−0.44
**8**	0.93	1.17			1.02		150	−0.34
**9**	0.66	0.91	1.14	0.82	1.07		160	−0.17
**10**		0.84			0.74		100	−0.16
**11**	0.79	1.21	1.34					
**12**		0.96			0.87		90	−0.36
**13**	0.75	0.99			0.90		90	−0.41
**14**	0.72	0.96			0.84		120	−0.32
**15**	0.80	1.10	1.50	0.70	0.95	1.35	100	−0.14
**16**	0.78	1.41	1.70					
**17**	0.81	1.53	1.62					

**Table 2:** ORAC-FL index of nitroisoxazoles derivatives.

Compound	ORAC-FL Index	Compound	ORAC-FL Index	Compound	ORAC-FL Index
**1**	0.9 ± 0.2	**7**	0.57 ± 0.08	**13**	0.4 ± 0.1
**2**	0.14 ± 0.05	**8**	1.0 ± 0.1	**14**	0.4 ± 0.1
**3**	0.52 ± 0.05	**9**	0.7 ± 0.2	**15**	0.8 ± 0.3
**4**	0.33 ± 0.06	**10**	0.36 ± 0.07	**16**	0.54 ± 0.07
**5**	0.74 ± 0.05	**11**	0.21 ± 0.01	**17**	0.4 ± 0.2
**6**	0.8 ± 0.1	**12**	0.4 ± 0.1	Trolox	1.00 ± 0.01

**Table 3:** Trypanocidal effect on epimastigote and trypomastigote of *T. cruzi* Dm28 by nitro isoxazoles derivates at 100 µM.

Compound	% *T. cruzi*		Log *p*	Compound	% *T. cruzi*		Log *p*
	Trypomastigote	Epimastigote			Trypomastigote	Epimastigote	
**1**	n.a ^[c]^	18 ± 1	1.12	**12**	16 ± 2	5 ± 2	5.61
**2**	n.a	19 ± 3	1.75	**13**	14 ± 3	n.a	6.38
**3**	20 ± 2	17 ± 5	2.31	**14**	3 ± 1	27 ± 2	6.11
**4**	0 ± 2	47 ± 4	3.07	**15**	n.a	29 ± 2	3.94
**5**	19 ± 3	47 ± 1	3.21	**16**	22 ± 6	19 ± 3	3.94
**6**	n.a	28 ± 2	3.95	**17**	15 ± 5	27 ± 1	3.94
**7**	n.a	36 ± 2	2.81	**P_1_** ^[b]^	n.a	14 ± 3	0.57
**8**	16 ± 2	32 ± 5	1.81	**P_2_** ^[b]^	n.a	5 ± 2	1.43
**9**	15 ± 3	52 ± 4	2.25	**Nfx** ^[a]^	74 ± 1	98.9 ± 0.3	
**10**	n.a	42 ± 2	2.25				
**11**	10 ± 4	n.a	4.07				

^[a]^ nifurtimox, ^[b]^ precursor: P_1_ 3,5-dimethyl-4-nitroisoxazole and P_2_: 4-bromophenyl acetaldehyde, ^[c]^ not active.

**Table 4:** IC_50_ values on the trypomastigote form of *T. cruzi* Dm28 and Vero mammalian cells.

Compound	*T. cruzi* (µM)		Vero Cells (µM)	SI ^[b]^	
	T ^[c]^	E ^[d]^		T ^[c]^	E ^[d]^
**3**	>400	>400	>400	nd ^[a]^	nd
**5**	>400	400 ± 3	>400	nd	nd
**8**	>400	> 400	398.1 ± 0.3	nd	nd
**9**	400 ± 2	166 ± 4	244.5 ± 0.5	0.6	1.5
**11**	400 ± 5	>400	125.9 ± 0.1	0.3	nd
**12**	>400	>400	141 ± 6	nd	nd
**13**	>400	>400	102 ± 6	nd	nd
**16**	>400	>400	141 ± 4	nd	nd
**17**	>400	>400	>400	nd	nd
**Nfx**	10.04 ± 0.1	-	172.9 ± 0.1	17.9	-

^[a]^ not define, ^[b]^ Selectivity index $SI=\frac{{IC}_{50}Vero\;cells}{{IC}_{50}\mathrm{T. cruzi}}$ , ^[c]^ trypomastigote, ^[d]^ epimastigote.

**Table 5:** Computational docking scores on cruzipain of *T. cruzi*.

Compound	Docking Score (kcal·mol^−1^)	Compound	Docking Score (kcal·mol^−1^)
**Native**	−10.03	**9**	−5.36
**1**	−4.13	**10**	−4.60
**2**	−4.58	**11**	−6.65
**3**	−4.82	**12**	−5.18
**4**	−4.90	**13**	−5.09
**5**	−4.77	**14**	−5.64
**6**	−4.91	**15**	−5.22
**7**	−4.29	**16**	−5.48
**8**	−5.09	**17**	−5.26

## Data Availability

Data are contained within the article and Supplementary Materials.

## Supplementary Materials

The following supporting information can be downloaded at: https://www.mdpi.com/article/10.3390/molecules29122762/s1, Figure S1: NMR spectra of compounds, Figure S2: Profile Physicochemical properties; Table S1: Hyperfine coupling constants of compounds **1**–**17** using ESR-electrochemical, Table S2: ADME parameters; Scheme S1: A reduction mechanism was proposed for compound **9** and compound **8**.
