# Supplementary material for: Assessment of the Activity of Nitroisoxazole Derivatives against Trypanosoma cruzi
Source: Molecules. 2024 Jun 11;29(12):2762. doi: 10.3390/molecules29122762 (PMC11207111; doi:10.3390/molecules29122762)

Supporting Information

Assessment of the Activity of Nitroisoxazole Derivatives against *Trypanosoma cruzi*

Mauricio Moncada-Basualto [1], Jorge Saavedra-Olavarría [2], Paula S. Rivero-Jerez [2], Cristian Rojas [1,3], Juan D. Maya [4], Ana Liempi [5], Matías Zúñiga-Bustos [1], Claudio Olea-Azar [3], Michel Lapier [6], Edwin G. Pérez [2,*] and Josué Pozo-Martínez [4,7,*]

[1] Instituto Universitario de Investigación y Desarrollo Tecnológico, Universidad Tecnológica Metropolitana, San Joaquín 8940577, Chile
[2] Department of Organic Chemistry, Faculty of Chemistry and Pharmacy, Pontificia Universidad Católica de Chile, Av. Vicuña Mackenna 4860, San Joaquin 7820436, Chile
[3] Laboratory of Free Radicals and Antioxidants, Faculty of Chemical and Pharmaceutical Science, Universidad de Chile, Olivos 1007, Independencia 8380544, Chile
[4] Programa de Farmacología Molecular y Clínica, Instituto de Ciencias Biomédicas, Facultad de Medicina, Universidad de Chile, Av. Independencia 1027, Independencia 8380453, Chile
[5] Programa de Biología Integrativa, Instituto de Ciencias Biomédicas, Facultad de Medicina, Universidad de Chile, Av. Independencia 1027, Independencia 8380453, Chile
[6] Centro de Investigación, Desarrollo e Innovación de Productos Bioactivos (CinBio), Escuela de Química y Farmacia, Facultad de Farmacia, Universidad de Valparaíso, Av. Gran Bretaña 1093, Valparaiso 2360102, Chile
[7] Laboratorio de Química—Médica, Facultad de Ciencia y Tecnología, Universidad del Azuay, Av. 24 de mayo 777, Cuenca 010204, Ecuador
* Correspondence: eperezh@uc.cl (E.G.P.); jspozom@yahoo.es (J.P.-M.)

Table S1. Hyperfine coupling constants of compounds **1–17** using ESR-electrochemical.

C	aN1/NO_2	aN2	aN3	aH1	aH2	aH2′	aH2′	aH3′	aH3′	aH4′	aF4′	a$F_3$4′	aBr
**1**	2.55	1.19	0.87	4.30	3.34								
**2**	4.08	1.43		5.01	2.05								
**3**	3.48	1.52	1.23	5.56	3.14								
**4**	4.74	1.24		5.37	1.93	0.71	0.71	0.50	0.50	0.66			
**5**	2.14	0.76		5.55	3.45	2.51	2.51	0.80	0.80		1.53		
**6**	4.74	0.92		6.15	3.19	1.77	1.77	1.36	1.36			0.83	
**7**	4.91	0.94	2.01	6.66	3.02	1.30	1.30	1.25	1.25				
**8**	2.31	0.82		5.68	3.45	2.63	1.11	1.06					
**9**	2.02	0.90		4.09	3.01	0.88	0.79	0.73					
**10**	4.36	1.59		5.18	2.22	0.71		0.49		0.20			
**11**	1.43	0.87	1.68	5.57	4.66	2.48*	1.83**	0.84***	0.92****				
**15**	2.27	2.30		3.43	3.41	1.20	1.22	1.10	1.10				3.91
**16**	2.29	2.27		3.51	3.48	1.24	1.20	0.94					3.95
**17**	2.30	2.38		3.56	3.49	1.18	1.18	1.10	1.10				3.99
	aN1/NO_2	aN2	aH1	aH2	Indole			Aril					aCl
					aN1′	aH2′	aH3′	aH2′	aH3′	aH4′	aH5′	aH6′	
**12**	3.38	2.50	3.90	3.85	2.51	2.54	1.49	1.00	1.53	1.51	1.53	1.00	
**13**	2.51	2.53	3.98	3.93	2.51	2.47	1.44	1.04	1.50		1.50	1.04	1.75
**14**	2.62	2.61	4.18	4.30	2.63	2.57	1.55	1.03	1.65		1.65	1.03	

C: compound. In the coumarin position: *4, ** 5, ***7 y **** 8

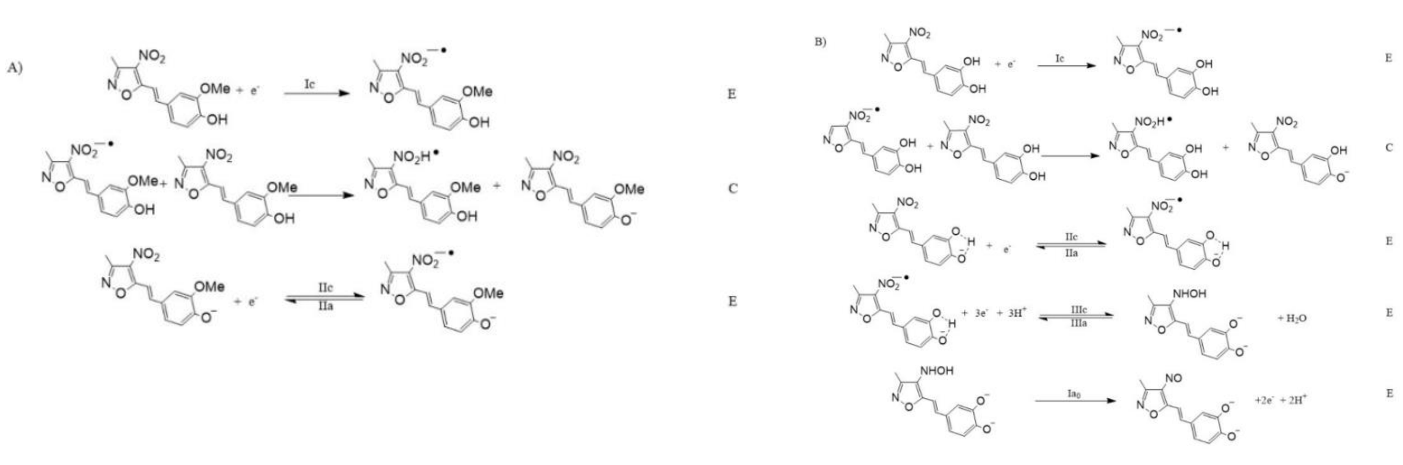

Scheme S1. A reduction mechanism was proposed for **A)** compound **9** and **B)** compound **8**, in both cases with labile hydrogens in their structure; electrochemical (**E**) and chemical (**C**) steps are indicated.

Table S2: ADME parameters.

C	Canonical SMILES	Formula	MW	#Heavy atoms	#Aromatic heavy atoms	Fraction Csp3	#Rotatable bonds	#H-bond acceptors	#H-bond donors
1	CN(/C=C/c1onc(c1[N+](=O)[O-])C)C	$C_8H_{11}N_3O_3$	197,19	14	5	0,38	3	4	0
2	Cc1noc(c1[N+](=O)[O-])/C=C/N1CCCC1	$C_{10}H_{13}N_3O_3$	223,23	16	5	0,5	3	4	0
3	[O-][N+](=O)c1c(/C=C/N2CCCCC2)onc1C	$C_{11}H_{15}N_3O_3$	237,26	17	5	0,55	3	4	0
4	[O-][N+](=O)c1c(C)noc1/C=C/c1ccccc1	$C_{12}H_{10}N_2O_3$	230,22	17	11	0,08	3	4	0
5	Fc1ccc(cc1)/C=C/c1onc(c1[N+](=O)[O-])C	$C_{12}H_9FN_2O_3$	248,21	18	11	0,08	3	5	0
6	[O-][N+](=O)c1c(/C=C/c2ccc(cc2)C(F)(F)F)onc1C	$C_{13}H_9F_3N_2O_3$	298,22	21	11	0,15	4	7	0
7	[O-][N+](=O)c1ccc(cc1)/C=C/c1onc(c1[N+](=O)[O-])C	$C_{12}H_9N_3O_5$	275,22	20	11	0,08	4	6	0
8	[O-][N+](=O)c1c(/C=C/c2ccc(c(c2)O)O)onc1C	$C_{12}H_{10}N_2O_5$	262,22	19	11	0,08	3	6	2
9	COc1cc(/C=C/c2onc(c2[N+](=O)[O-])C)ccc1O	$C_{13}H_{12}N_2O_5$	276,24	20	11	0,15	4	6	1
10	[O-][N+](=O)c1c(/C=C/c2ccco2)onc1C	$C_{10}H_8N_2O_4$	220,18	16	10	0,1	3	5	0
11	CCN(c1ccc2c(c1)oc(=O)c(c2)/C=C/c1onc(c1[N+](=O)[O-])C)CC	$C_{19}H_{19}N_3O_5$	369,37	27	15	0,26	6	6	0
12	[O-][N+](=O)c1c(/C=C/c2cn(c3c2cccc3)c2ccccc2)onc1C	$C_{20}H_{15}N_3O_3$	345,35	26	20	0,05	4	4	0
13	Clc1ccc(cc1)n1cc(c2c1cccc2)/C=C/c1onc(c1[N+](=O)[O-])C	$C_{20}H_{14}ClN_3O_3$	379,8	27	20	0,05	4	4	0
14	Cc1ccc(cc1)n1cc(c2c1cccc2)/C=C/c1onc(c1[N+](=O)[O-])C	$C_{21}H_{17}N_3O_3$	359,38	27	20	0,1	4	4	0
15	Brc1ccccc1/C=C/c1onc(c1[N+](=O)[O-])C	$C_{12}H_9BrN_2O_3$	309,12	18	11	0,08	3	4	0
16	Brc1cccc(c1)/C=C/c1onc(c1[N+](=O)[O-])C	$C_{12}H_9BrN_2O_3$	309,12	18	11	0,08	3	4	0
17	Brc1ccc(cc1)/C=C/c1onc(c1[	$C_{12}H_9BrN_2O_3$	309,12	18	11	0,08	3	4	0

	N+](=O)[O-])C								

C	MR	TPSA	iLOGP	XLOGP3	WLOGP	MLOGP	Silicos-IT Log P	Consensus Log P	ESOL Log S
1	52,89	75,09	1,84	1,18	1,31	-0,59	-0,85	0,58	-1,87
2	64,31	75,09	2,16	1,67	1,47	0,03	-0,04	1,06	-2,31
3	69,11	75,09	2,39	2,03	1,86	0,32	0,2	1,36	-2,61
4	65,66	71,85	2,2	2,9	2,84	1,17	1,18	2,06	-3,38
5	65,62	71,85	2,23	3	3,4	1,58	1,6	2,36	-3,52
6	70,66	71,85	2,49	3,79	5,01	2,1	2,25	3,13	-4,2
7	74,48	117,67	1,93	2,73	2,75	0,25	-0,96	1,34	-3,41
8	69,71	112,31	1,71	2,19	2,26	0,1	0,23	1,3	-3,08
9	74,18	101,31	2,38	2,52	2,56	0,37	0,75	1,72	-3,28
10	57,93	84,99	2,01	2	2,44	-0,17	0,6	1,37	-2,73
11	105,52	105,3	3,16	3,76	3,8	1,61	2,15	2,9	-4,51
12	102,49	76,78	3,11	4,64	4,79	2,51	2,29	3,47	-5,21
13	107,5	76,78	3,36	5,27	5,44	3	2,92	4	-5,8
14	107,46	76,78	3,23	5	5,1	2,73	2,81	3,77	-5,5
15	73,36	71,85	2,51	3,59	3,61	1,84	1,86	2,68	-4,27
16	73,36	71,85	2,58	3,59	3,61	1,84	1,86	2,7	-4,27
17	73,36	71,85	2,5	3,59	3,61	1,84	1,86	2,68	-4,27

C	ESOL Solubility (mg/ml)	ESOL Solubility (mol/l)	ESOL Class	Ali Log S	Ali Solubility (mg/ml)	Ali Solubility (mol/l)	Ali Class	Silicos-IT LogSw	Silicos-IT Solubility (mg/ml)	Silicos-IT Solubility (mol/l)	Silicos-IT class
1	2.65e+00	1.34e-02	Very soluble	-2,35	8.76e-01	4.44e-03	Soluble	-1,21	1.21e+01	6.14e-02	Soluble
2	1.09e+00	4.90e-03	Soluble	-2,86	3.07e-01	1.38e-03	Soluble	-1,55	6.29e+00	2.82e-02	Soluble
3	5.83e-01	2.46e-03	Soluble	-3,23	1.38e-01	5.83e-04	Soluble	-1,83	3.53e+00	1.49e-02	Soluble
4	9.70e-02	4.22e-04	Soluble	-4,07	1.96e-02	8.52e-05	Moderately soluble	-3,62	5.51e-02	2.39e-04	Soluble
5	7.44e-02	3.00e-04	Soluble	-4,17	1.67e-02	6.71e-05	Moderately soluble	-3,9	3.14e-02	1.26e-04	Soluble
6	1.88e-02	6.31e-05	Moderately soluble	-4,99	3.03e-03	1.02e-05	Moderately soluble	-4,49	9.65e-03	3.24e-05	Moderately soluble
7	1.07e-01	3.90e-04	Solubl	-4,86	3.84e-03	1.40e-05	Moderat	-2,99	2.80e-01	1.02e-03	Soluble

			e				ely soluble				
8	2.20e-01	8.40e-04	Soluble	-4,18	1.72e-02	6.57e-05	Moderately soluble	-2,47	8.97e-01	3.42e-03	Soluble
9	1.44e-01	5.21e-04	Soluble	-4,29	1.40e-02	5.08e-05	Moderately soluble	-3,17	1.89e-01	6.84e-04	Soluble
10	4.10e-01	1.86e-03	Soluble	-3,41	8.54e-02	3.88e-04	Soluble	-2,83	3.22e-01	1.46e-03	Soluble
11	1.13e-02	3.06e-05	Moderately soluble	-5,66	8.00e-04	2.17e-06	Moderately soluble	-5,69	7.47e-04	2.02e-06	Moderately soluble
12	2.13e-03	6.17e-06	Moderately soluble	-5,98	3.63e-04	1.05e-06	Moderately soluble	-6,15	2.42e-04	7.01e-07	Poorly soluble
13	6.03e-04	1.59e-06	Moderately soluble	-6,63	8.86e-05	2.33e-07	Poorly soluble	-6,75	6.83e-05	1.80e-07	Poorly soluble
14	1.13e-03	3.15e-06	Moderately soluble	-6,35	1.60e-04	4.45e-07	Poorly soluble	-6,53	1.06e-04	2.94e-07	Poorly soluble
15	1.65e-02	5.34e-05	Moderately soluble	-4,79	5.07e-03	1.64e-05	Moderately soluble	-4,45	1.10e-02	3.55e-05	Moderately soluble
16	1.65e-02	5.34e-05	Moderately soluble	-4,79	5.07e-03	1.64e-05	Moderately soluble	-4,45	1.10e-02	3.55e-05	Moderately soluble
17	1.65e-02	5.34e-05	Moderately soluble	-4,79	5.07e-03	1.64e-05	Moderately soluble	-4,45	1.10e-02	3.55e-05	Moderately soluble

C	GI absorption	BBB permeant	Pgp substrate	CYP1A2 inhibitor	CYP2C19 inhibitor	CYP2C9 inhibitor	CYP2D6 inhibitor	CYP3A4 inhibitor
1	High	No	No	Yes	No	No	No	No
2	High	No	No	Yes	Yes	No	No	No
3	High	No	No	Yes	Yes	No	No	No
4	High	Yes	No	Yes	Yes	No	No	No
5	High	Yes	No	Yes	Yes	No	No	No
6	High	No	No	Yes	Yes	Yes	No	No
7	High	No	No	Yes	Yes	Yes	No	No
8	High	No	No	No	No	No	No	No
9	High	No	No	Yes	Yes	Yes	No	No
10	High	No	No	Yes	No	No	No	No
11	High	No	No	Yes	Yes	Yes	No	Yes
12	High	No	No	Yes	Yes	Yes	No	No
13	High	No	No	Yes	Yes	Yes	No	No
14	High	No	No	Yes	Yes	Yes	No	No
15	High	Yes	No	Yes	Yes	Yes	No	No
16	High	Yes	No	Yes	Yes	Yes	No	No
17	High	Yes	No	Yes	Yes	Yes	No	No

C	log Kp (cm/s)	Lipinski #violations	Ghose #violations	Veber #violations	Egan #violations	Muegge #violations	Bioavailability Score	PAINS #alerts	Brenk #alerts	Leadlikeness #violations	Synthetic Accessibility
1	-6,67	0	0	0	0	1	0,55	0	2	1	3,34
2	-6,48	0	0	0	0	0	0,55	0	2	1	3,34
3	-6,31	0	0	0	0	0	0,55	0	2	1	3,38
4	-5,65	0	0	0	0	0	0,55	0	2	1	3,19
5	-5,68	0	0	0	0	0	0,55	0	2	1	3,16
6	-5,43	0	0	0	0	0	0,55	0	2	1	3,28
7	-6,04	0	0	0	0	0	0,55	0	2	0	3,17
8	-6,34	0	0	0	0	0	0,55	1	3	0	3,17
9	-6,2	0	0	0	0	0	0,55	0	2	0	3,22
10	-6,22	0	0	0	0	0	0,55	0	2	1	3,22
11	-5,88	0	0	0	0	0	0,55	0	3	2	3,93
12	-5,11	0	0	0	0	0	0,55	0	2	1	3,52
13	-4,88	0	0	0	0	1	0,55	0	2	2	3,49
14	-4,94	0	0	0	0	0	0,55	0	2	2	3,62
15	-5,64	0	0	0	0	0	0,55	0	2	1	3,25
16	-5,64	0	0	0	0	0	0,55	0	2	1	3,21
17	-5,64	0	0	0	0	0	0,55	0	2	1	3,18

Figure S1: NMR Spectra of compounds

^{1}H-NMR spectrum of **1**

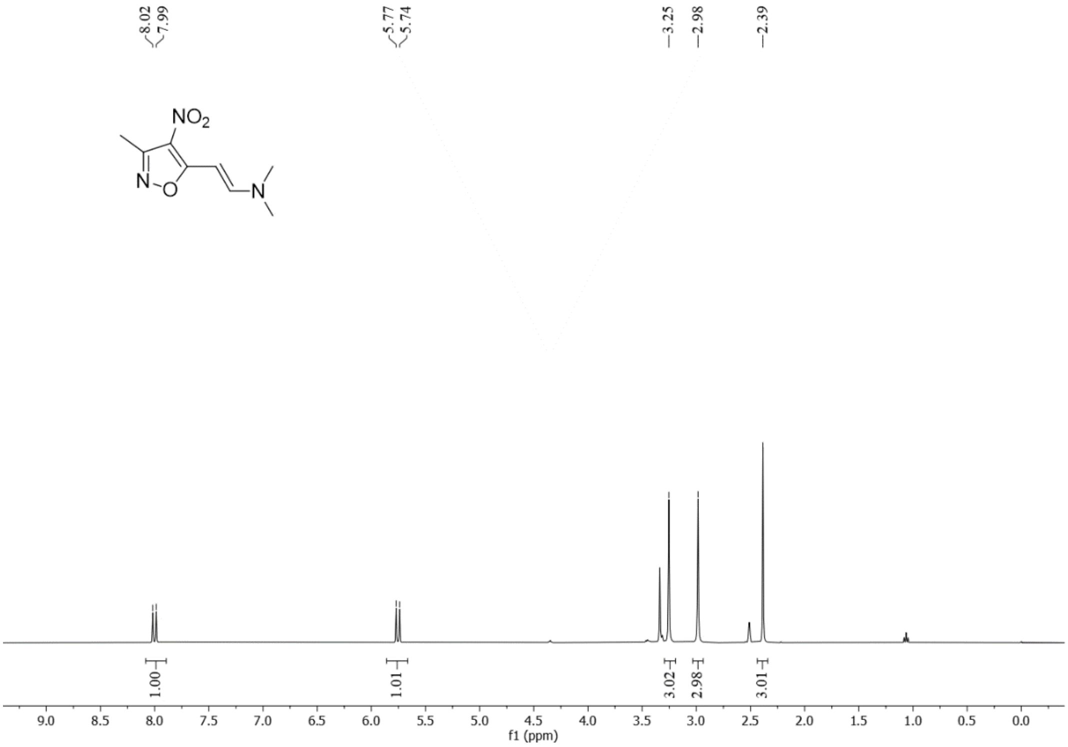

^{13}C-NMR spectrum of **1**

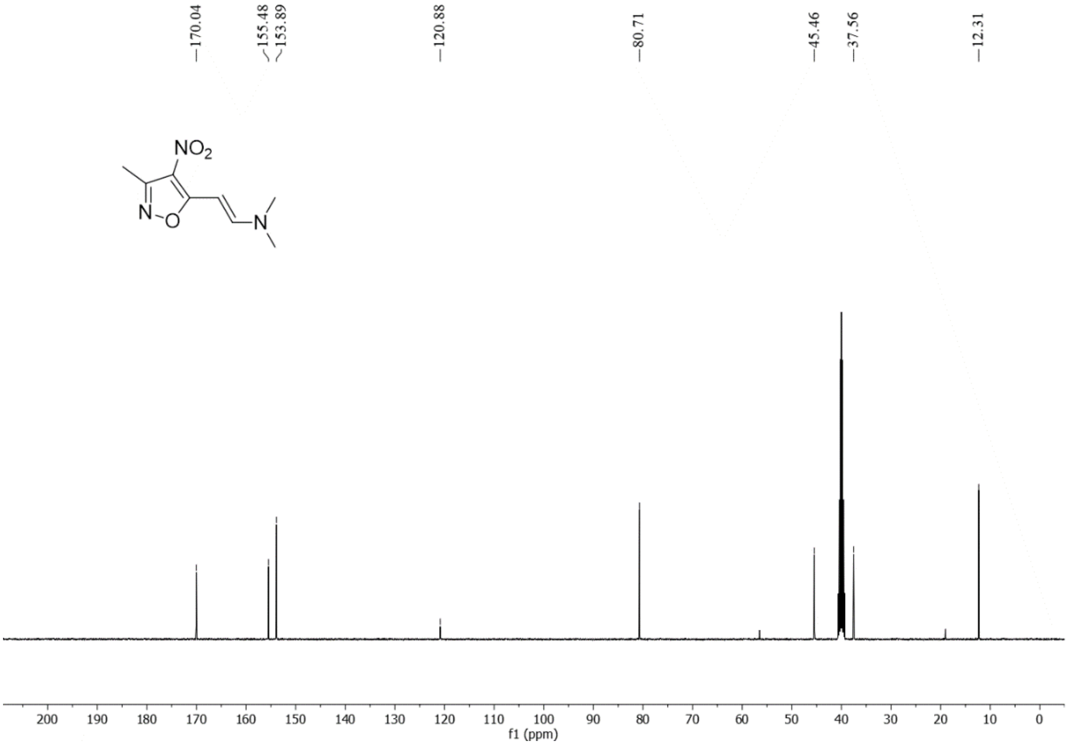

^{1}H-NMR spectrum of **2**

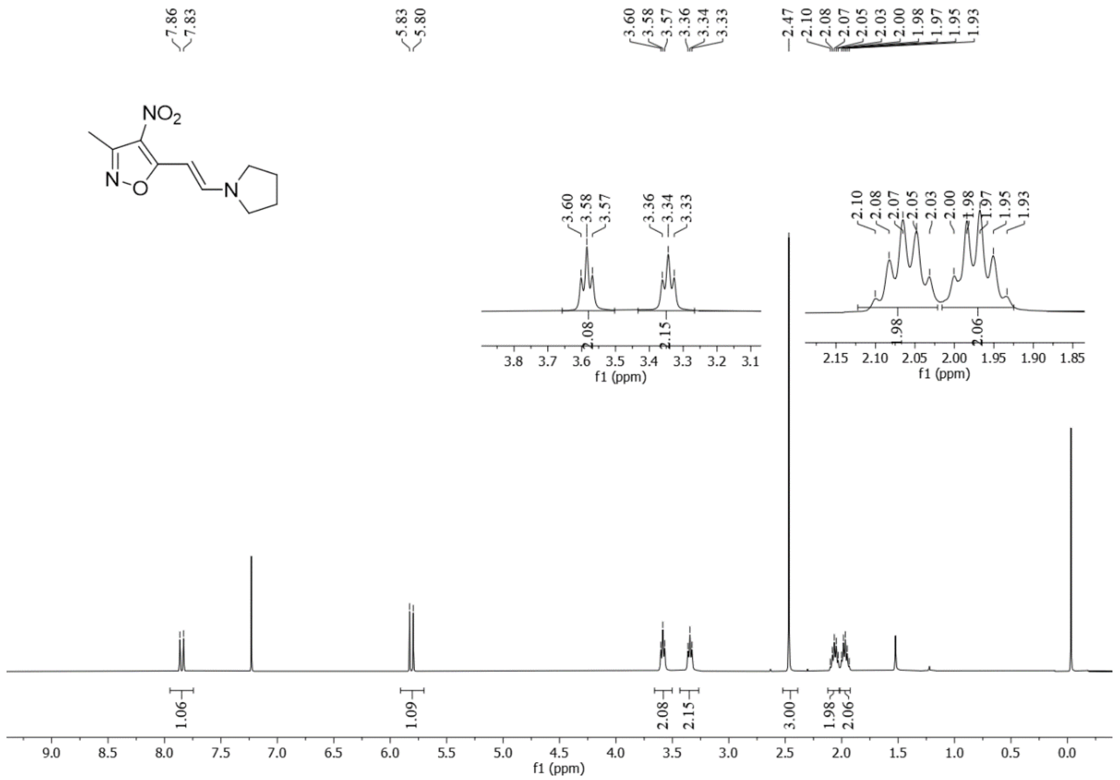

^{13}C-NMR spectrum of **2**

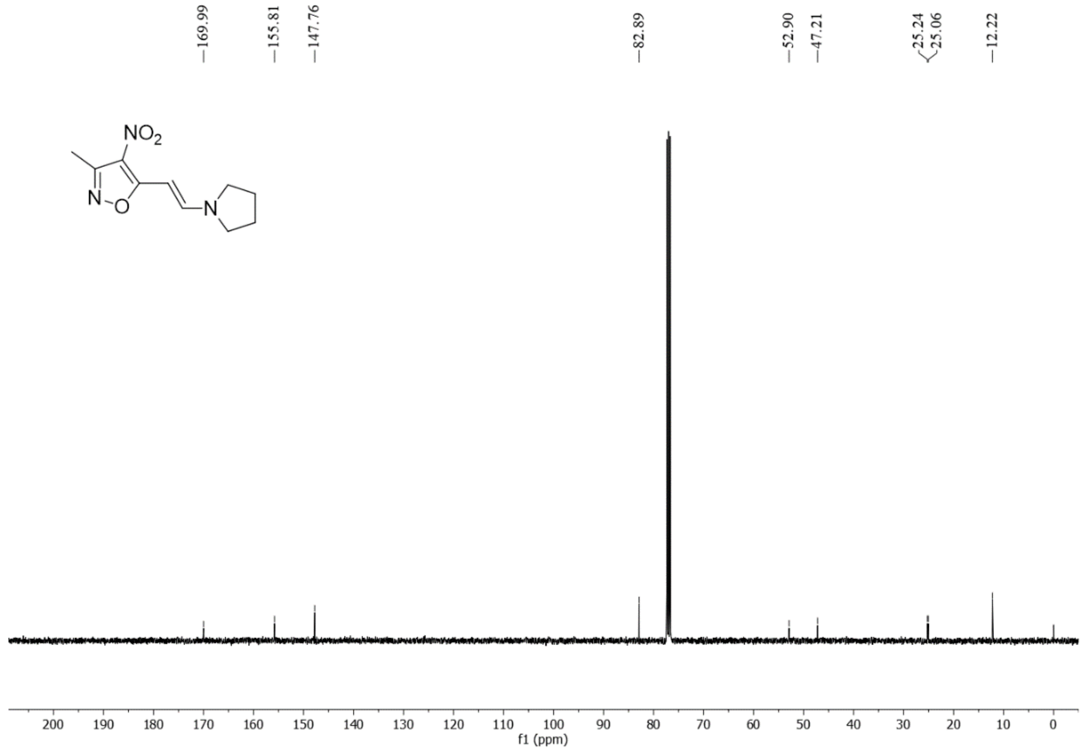

¹H-NMR spectrum of **3**

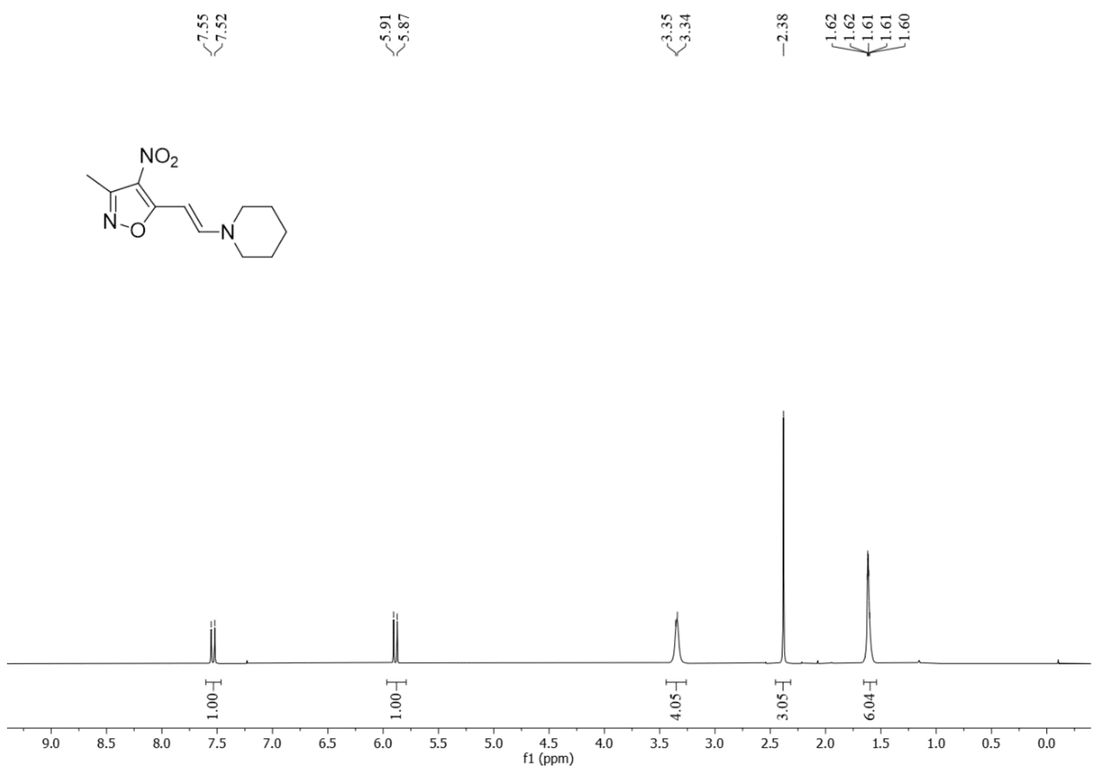

¹³C-NMR spectrum of **3**

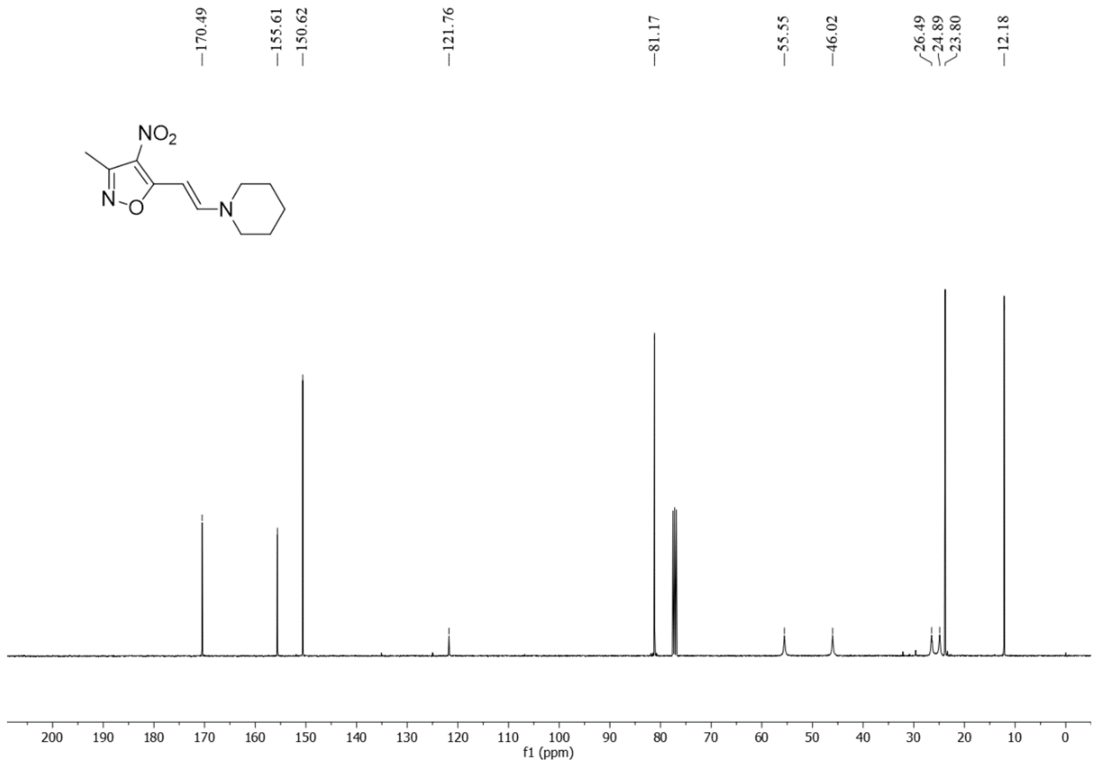

^{1}H-NMR spectrum of **4**

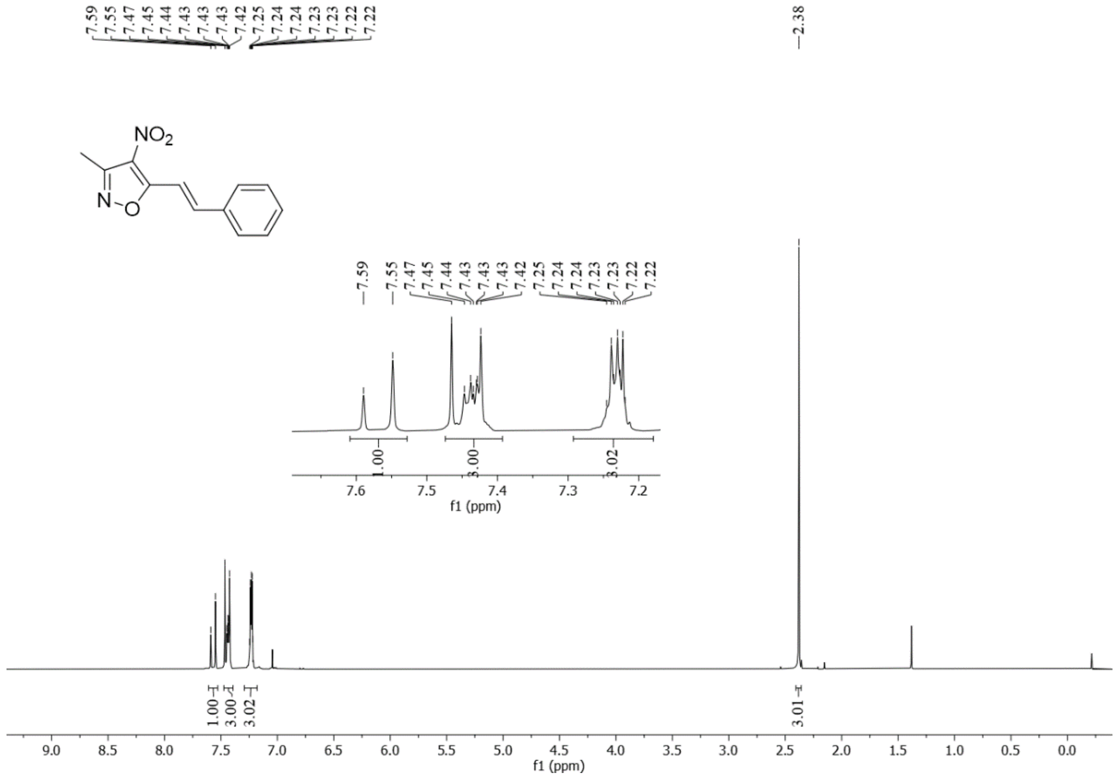

^{13}C-NMR spectrum of **4**

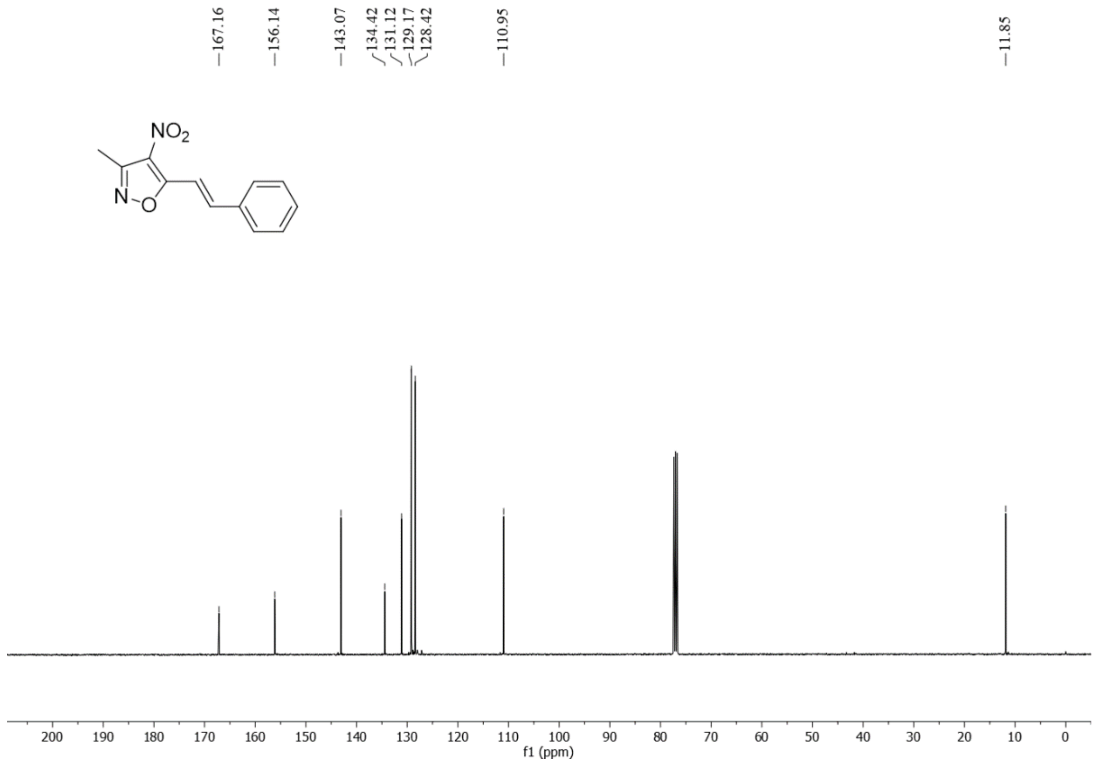

^{1}H-NMR spectrum of **5**

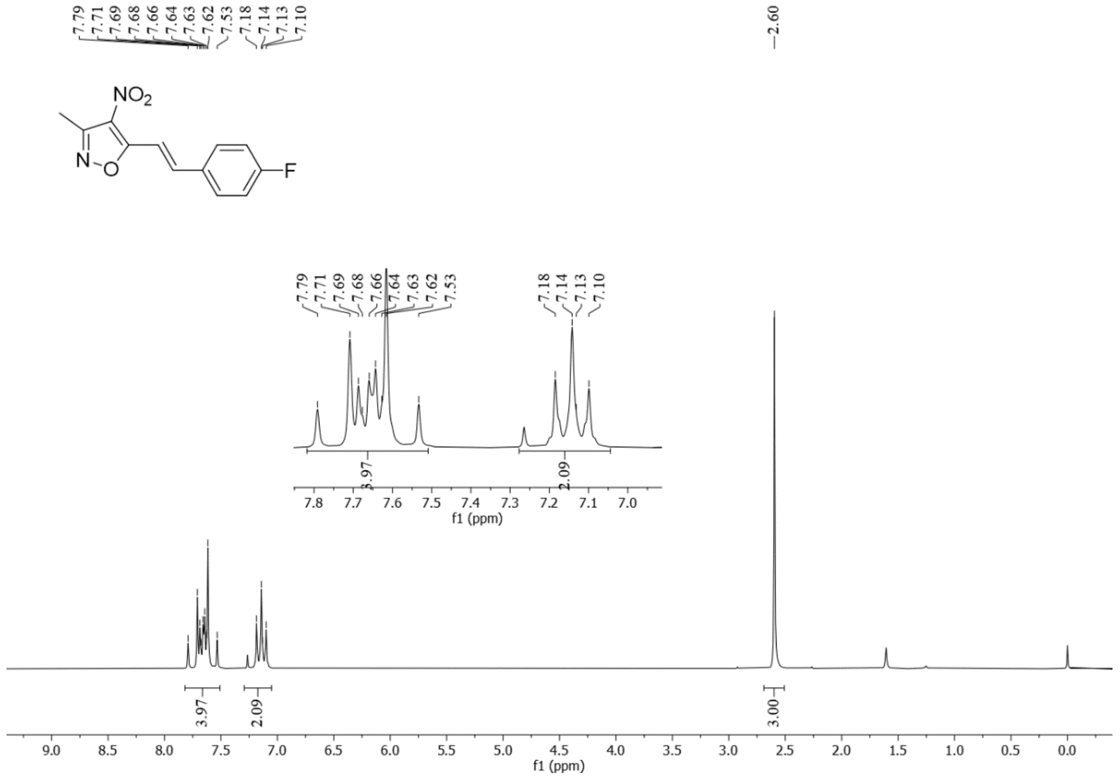

^{13}C-NMR spectrum of **5**

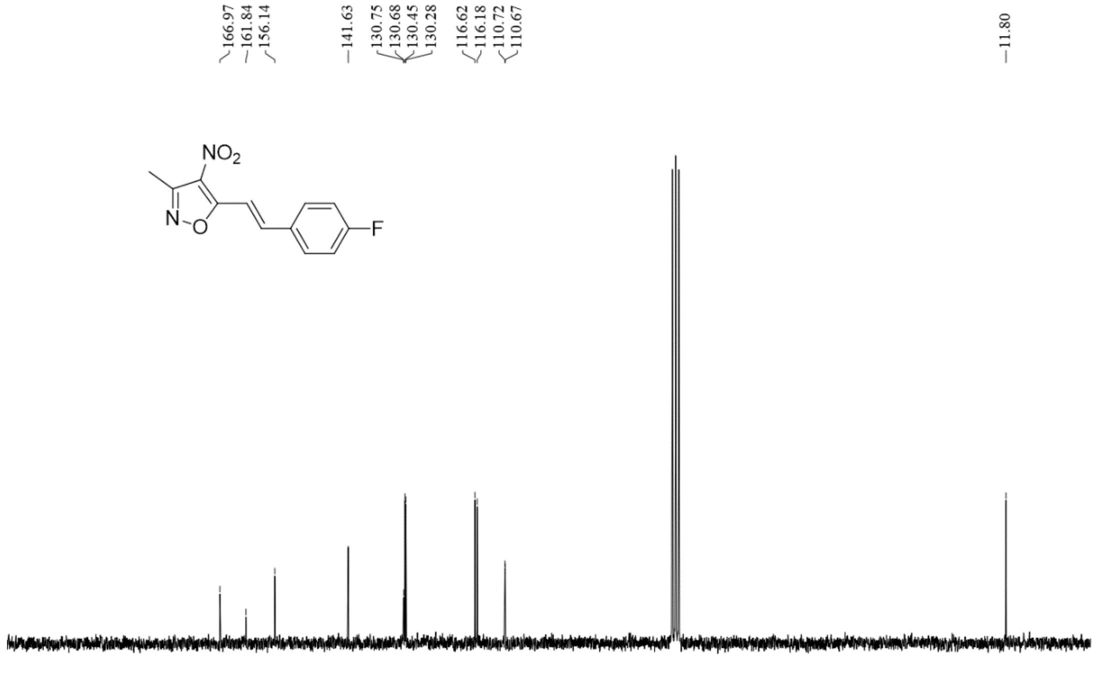

^{19}F-NMR spectrum of **5**

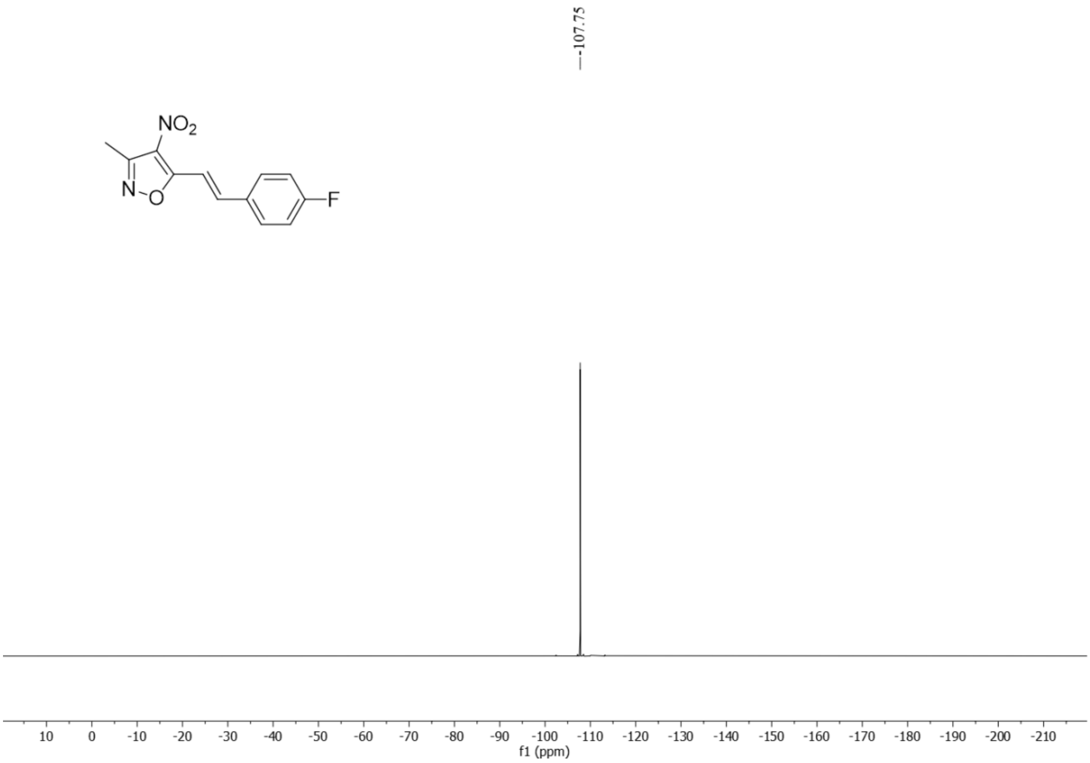

^{1}H-NMR spectrum of **6**

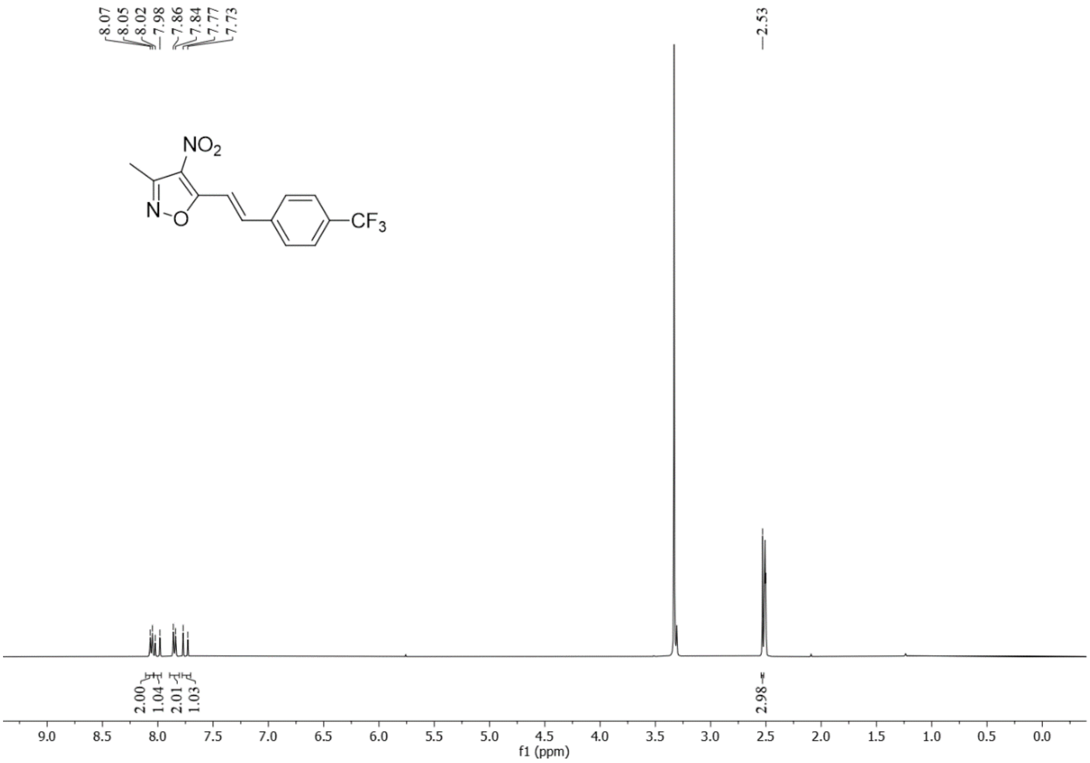

^{13}C-NMR spectrum of **6**

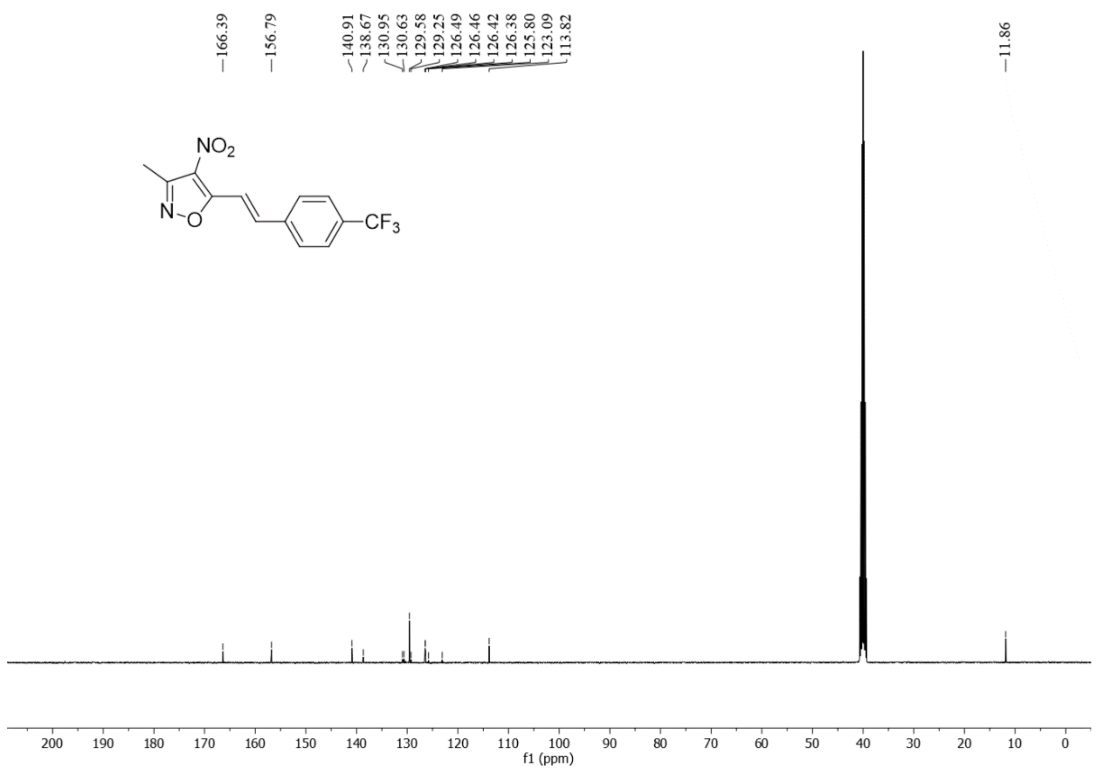

^{19}F-NMR spectrum of **6**

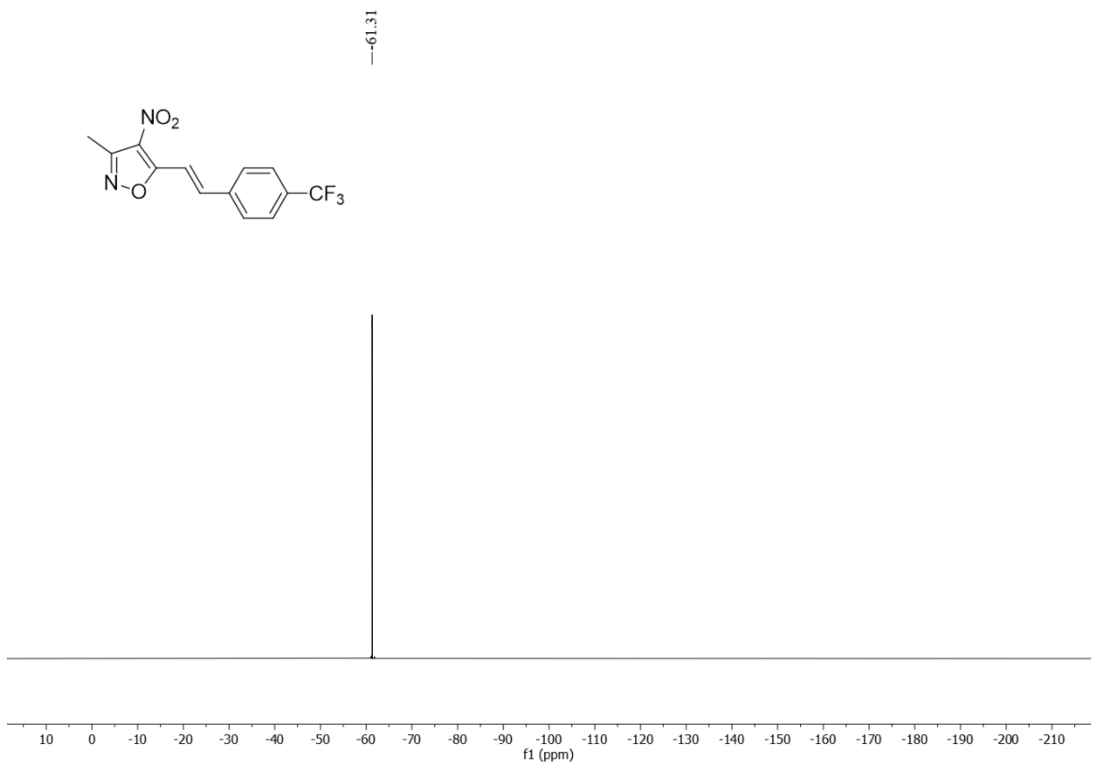

^{1}H-NMR spectrum of **7**

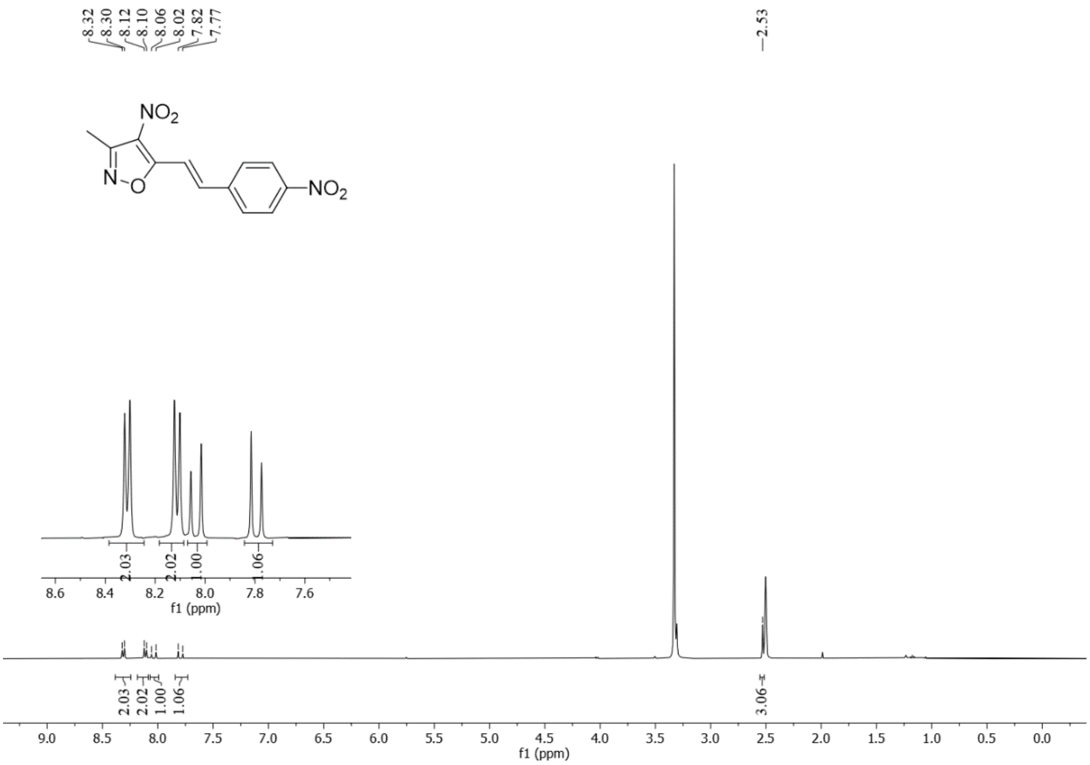

^{13}C-NMR spectrum of **7**

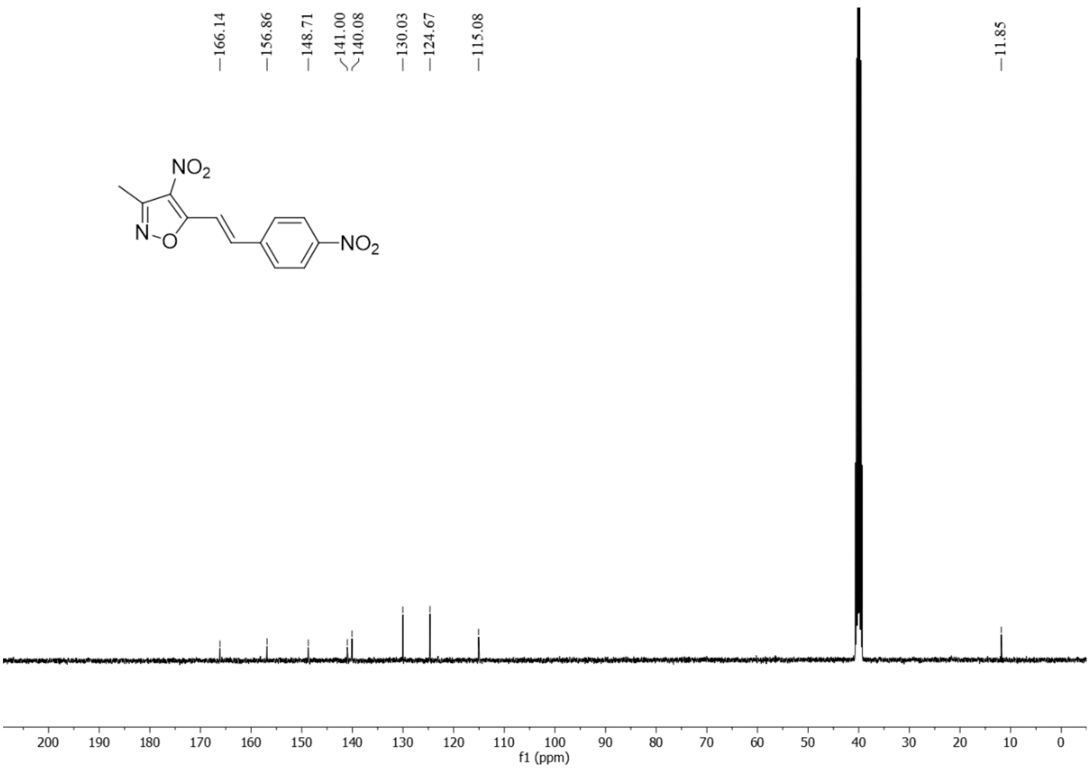

^{1}H-NMR spectrum of **8**

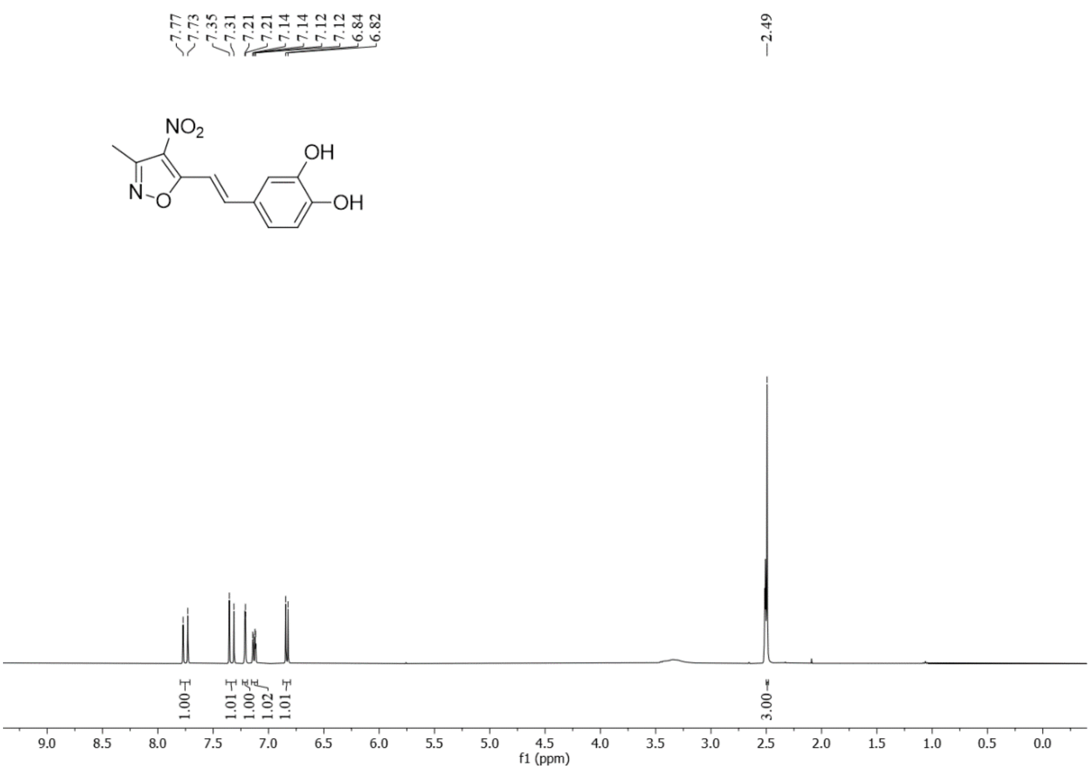

^{13}C-NMR spectrum of **8**

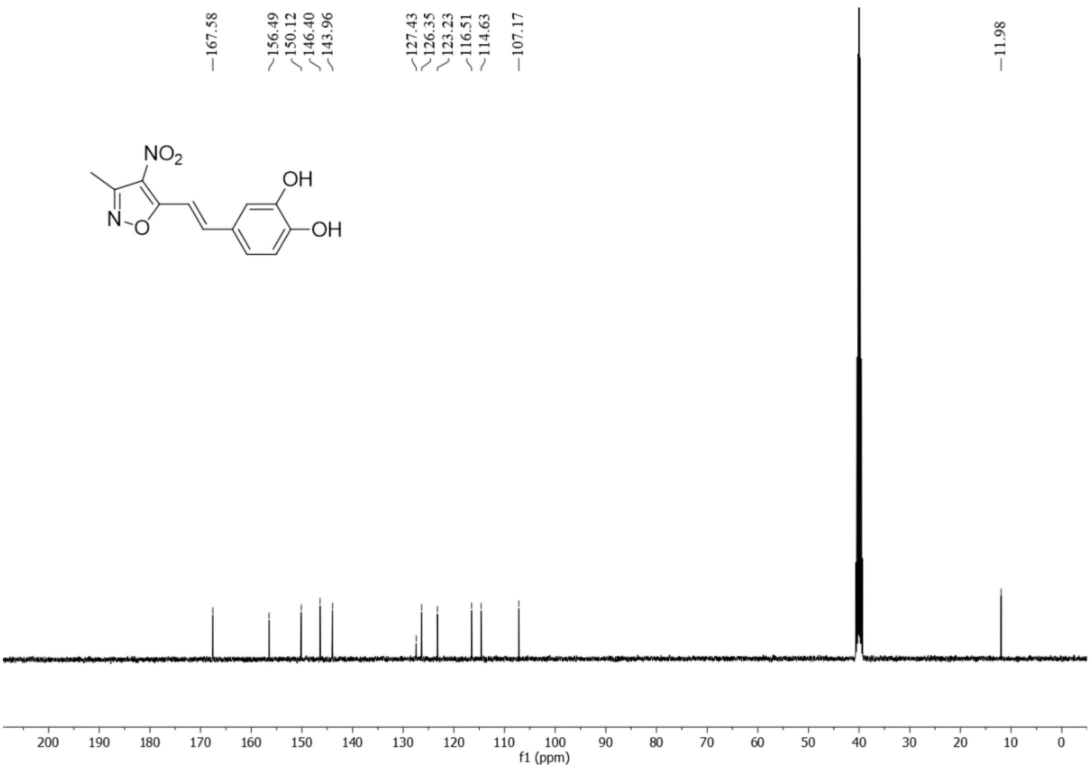

^{1}H-NMR spectrum of **9**

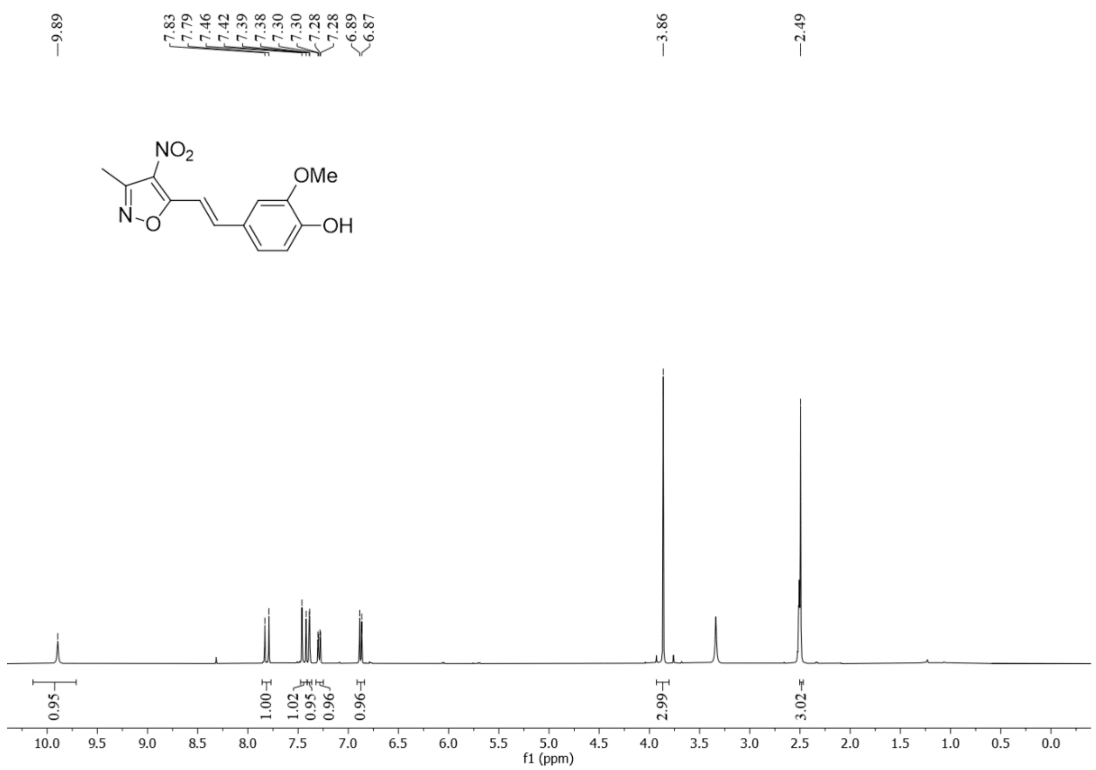

^{13}C-NMR spectrum of **9**

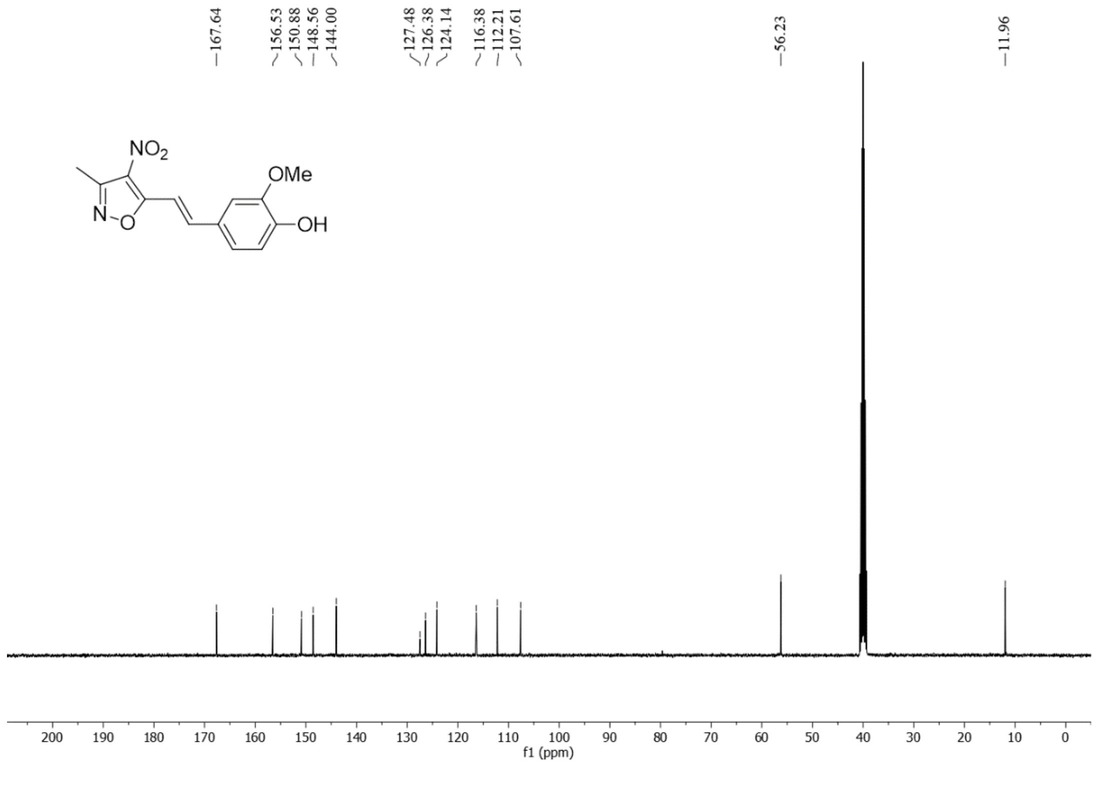

^{1}H-NMR spectrum of **10**

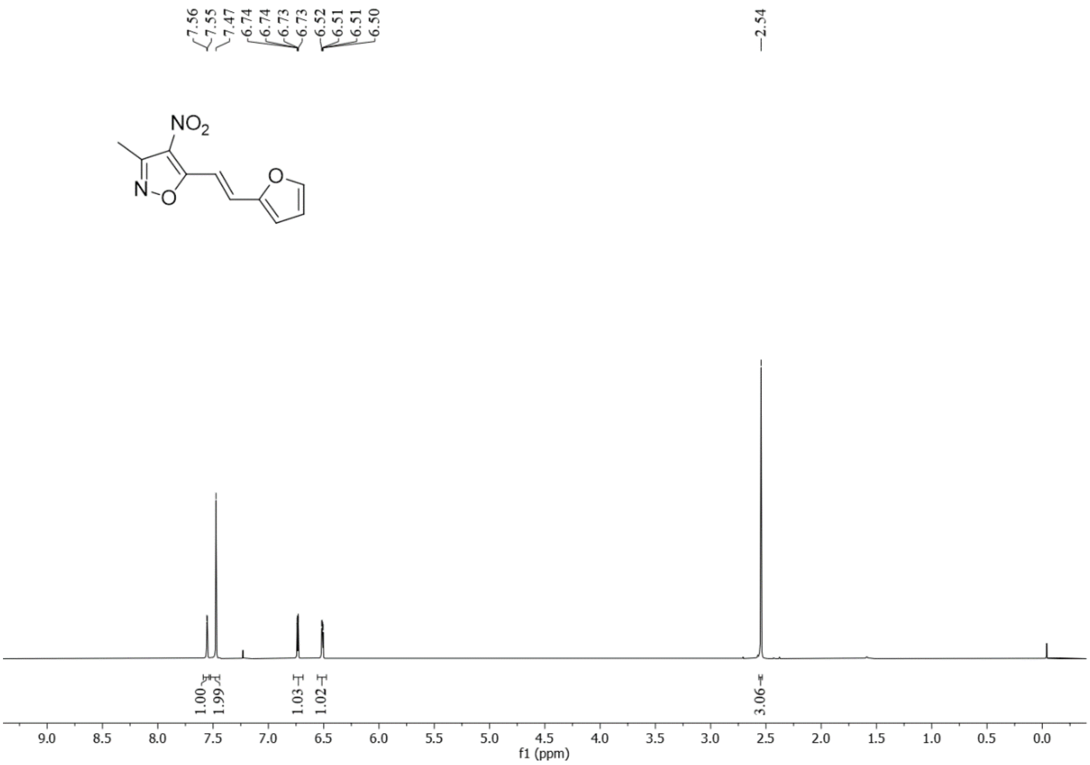

^{13}C-NMR spectrum of **10**

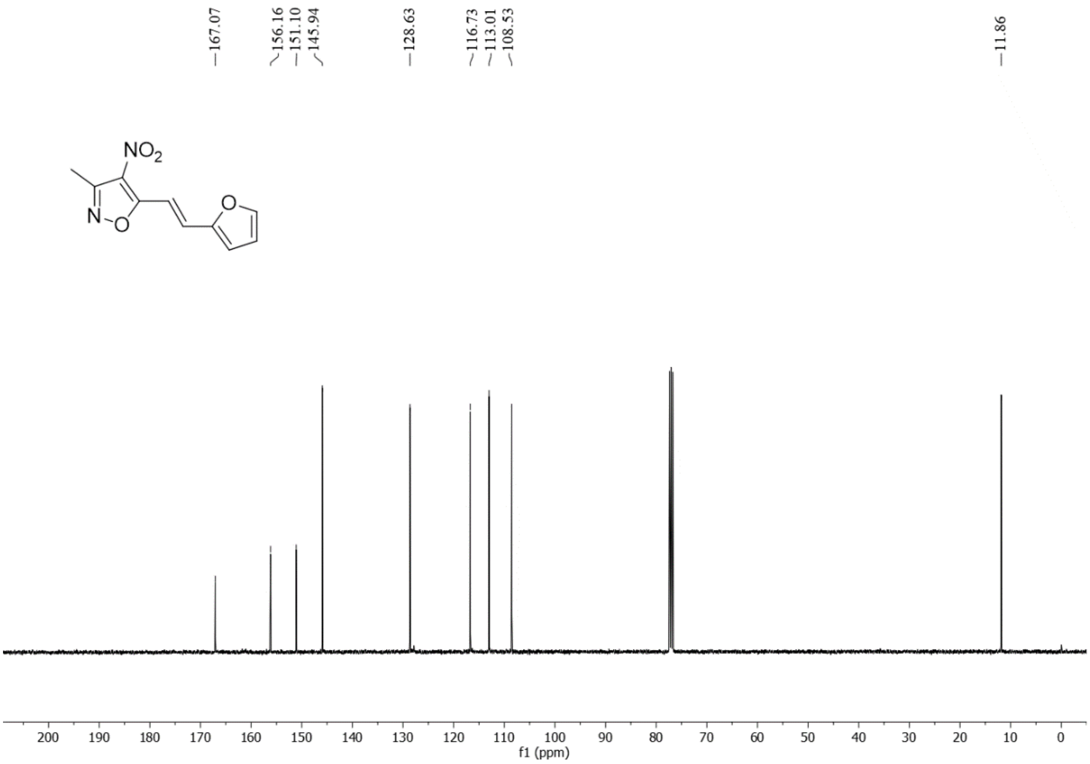

^{1}H-NMR spectrum of **11**

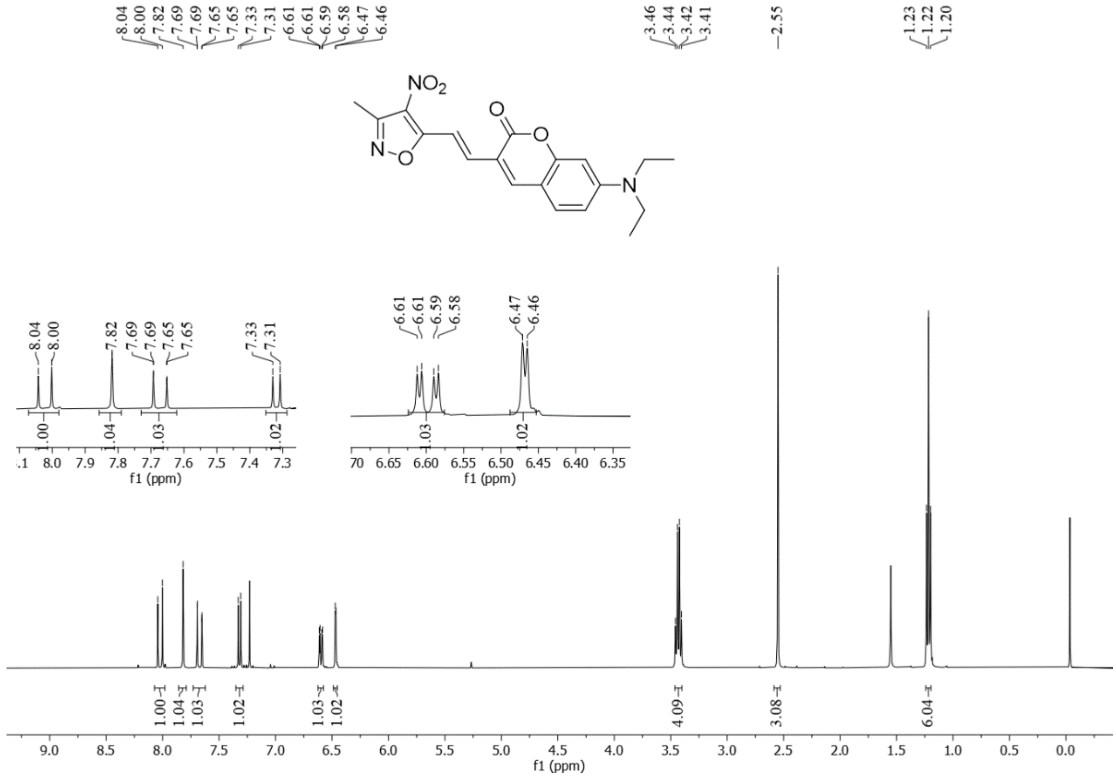

^{13}C-NMR spectrum of **11**

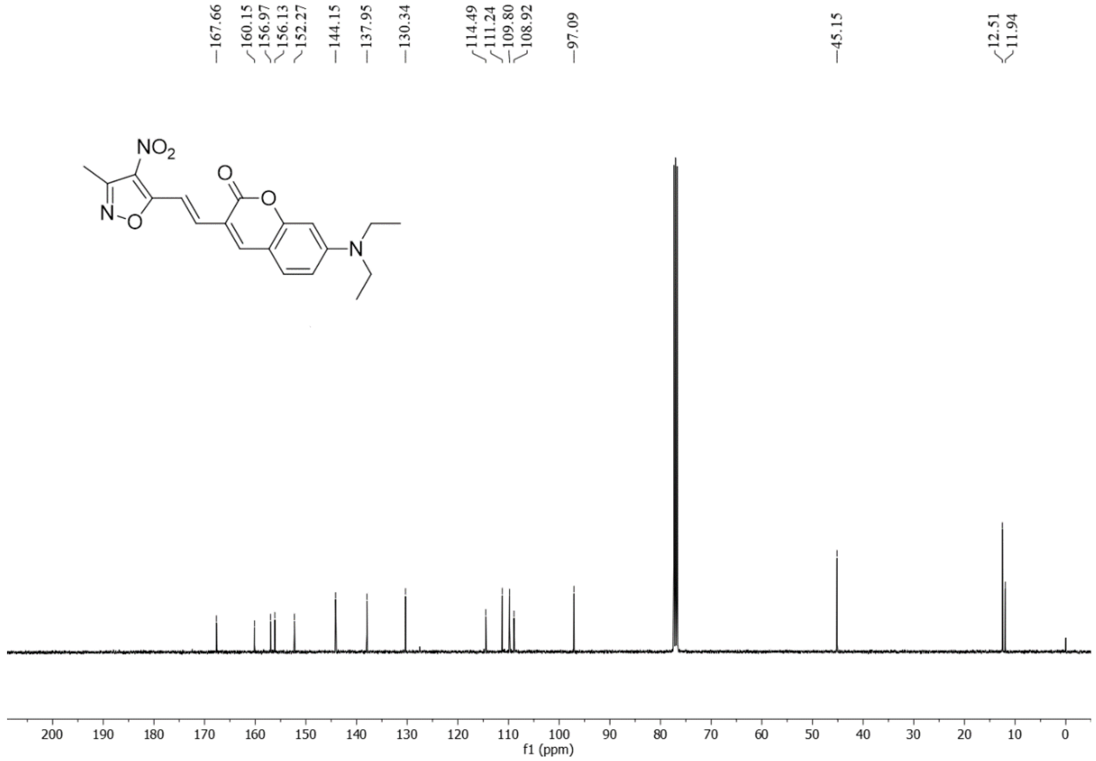

^{1}H-NMR spectrum of **12**

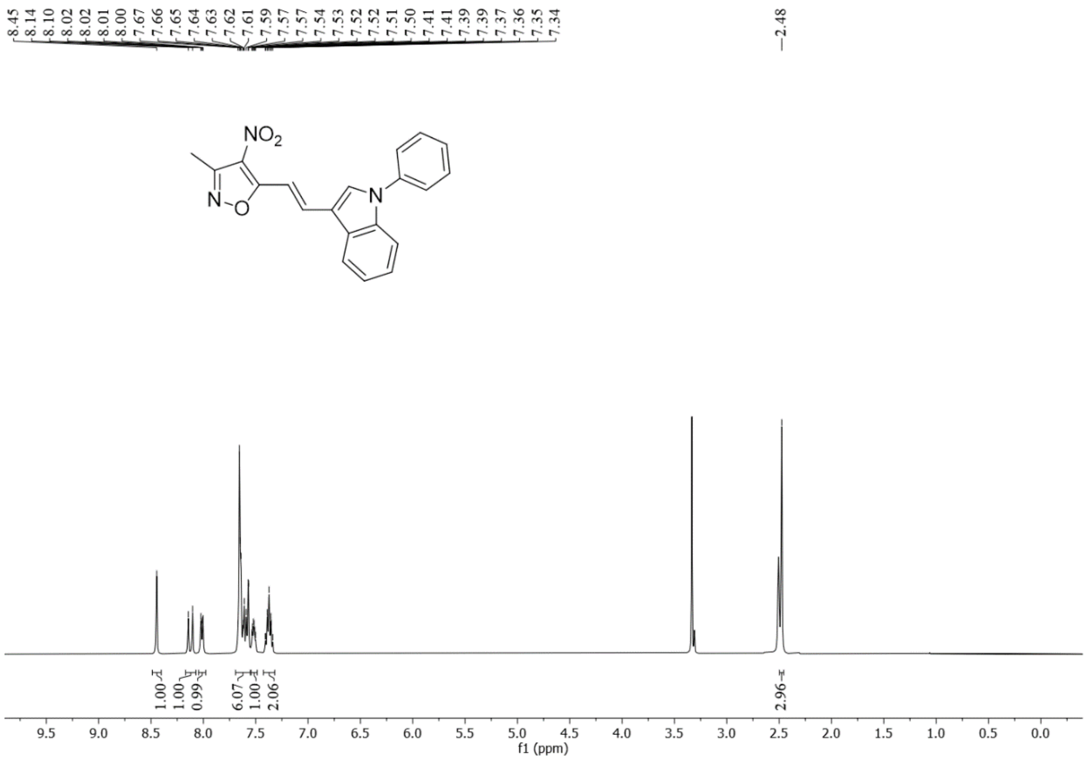

^{13}C-NMR spectrum of **12**

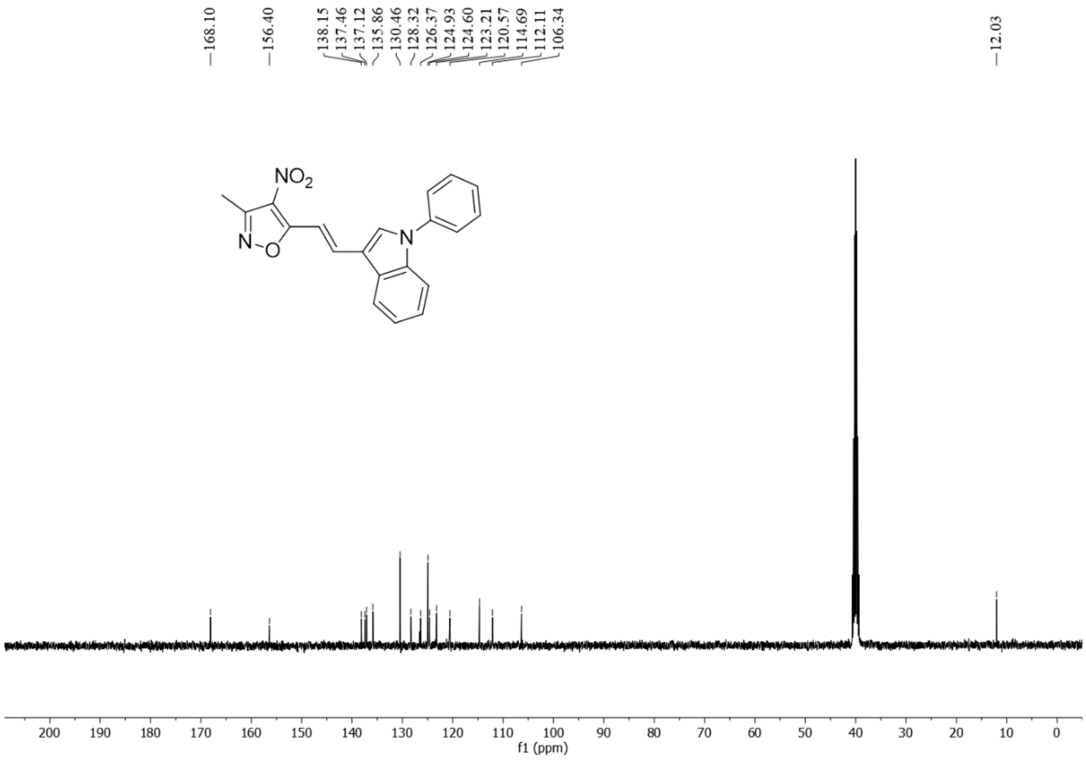

^{1}H-NMR spectrum of **13**

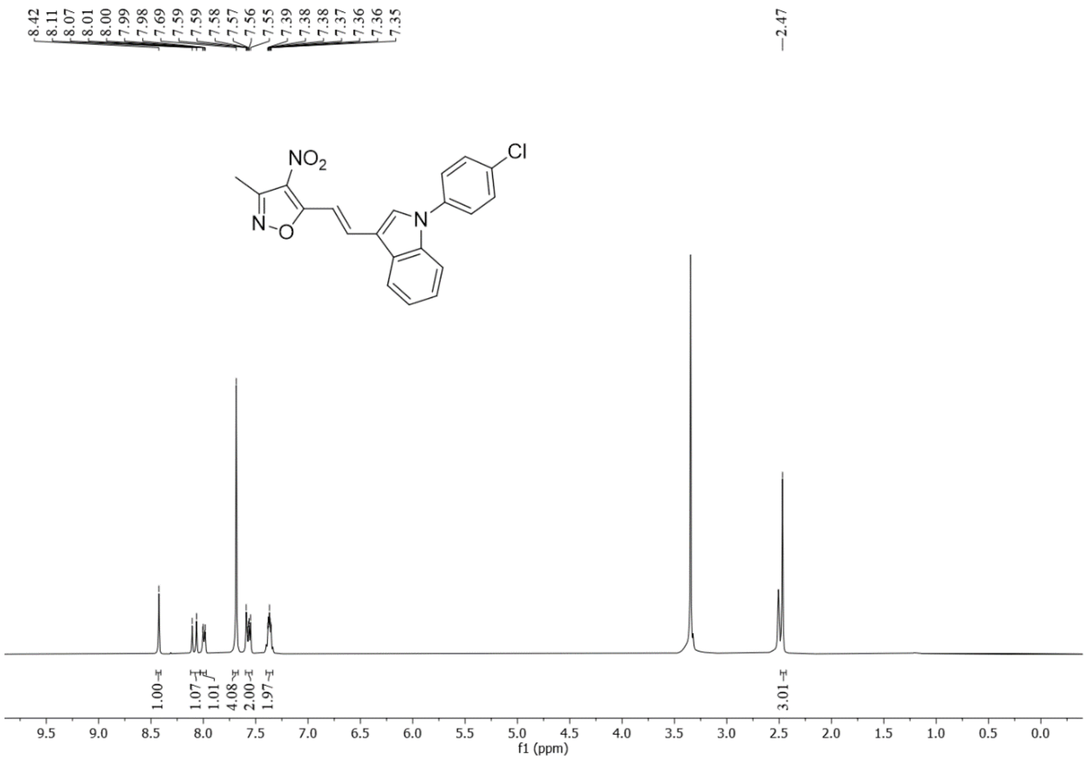

^{13}C-NMR spectrum of **13**

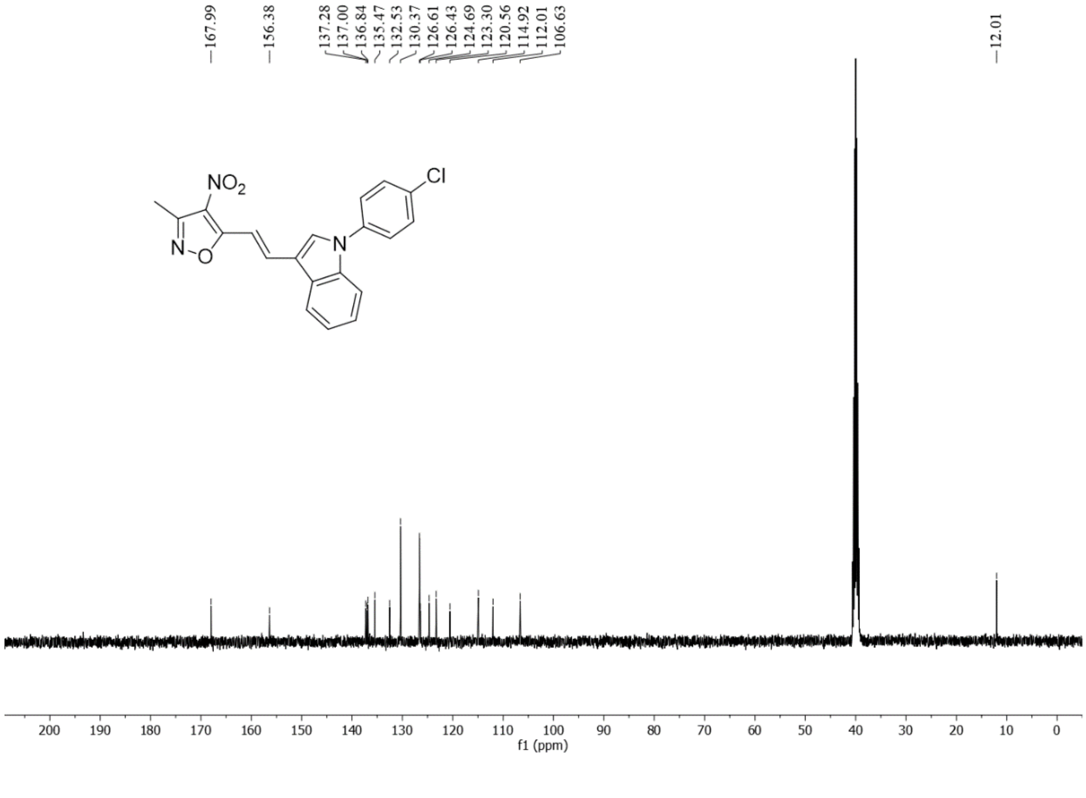

^{1}H-NMR spectrum of **14**

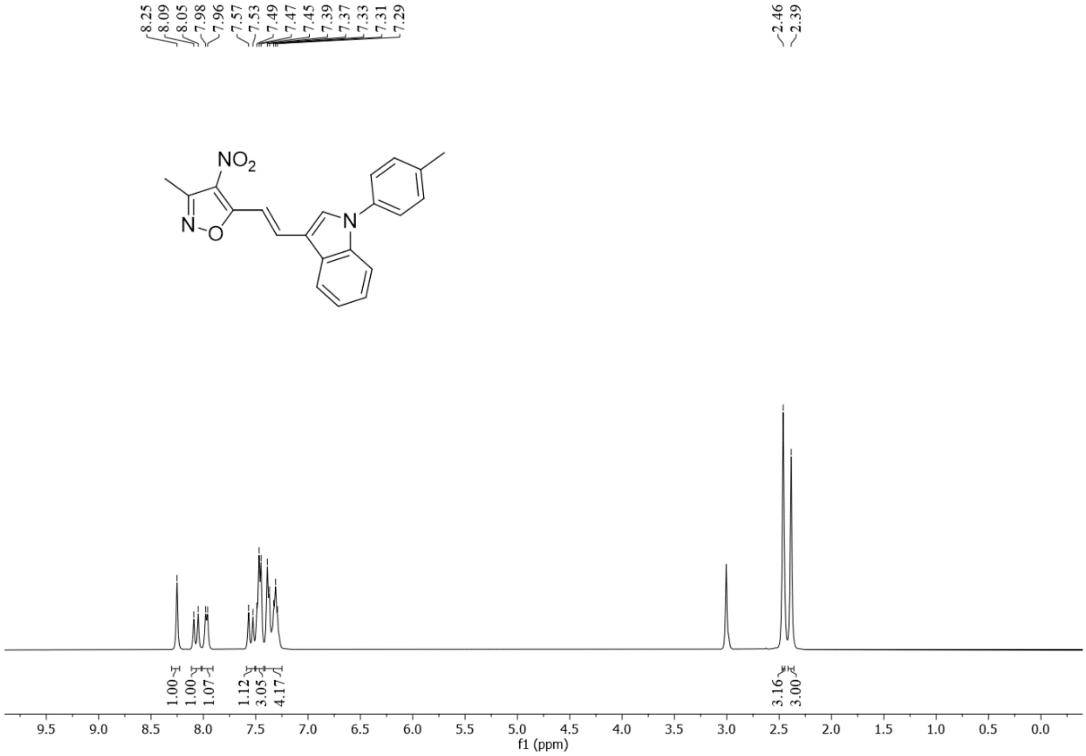

^{13}C-NMR spectrum of **14**

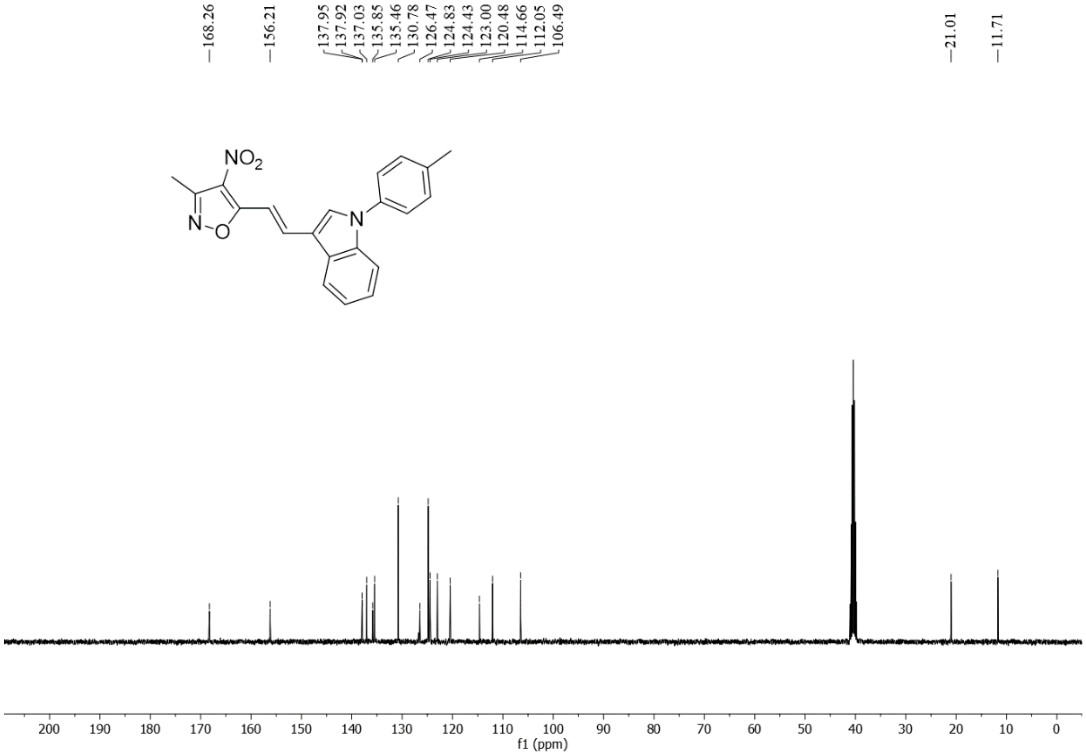

^{1}H-NMR spectrum of **15**

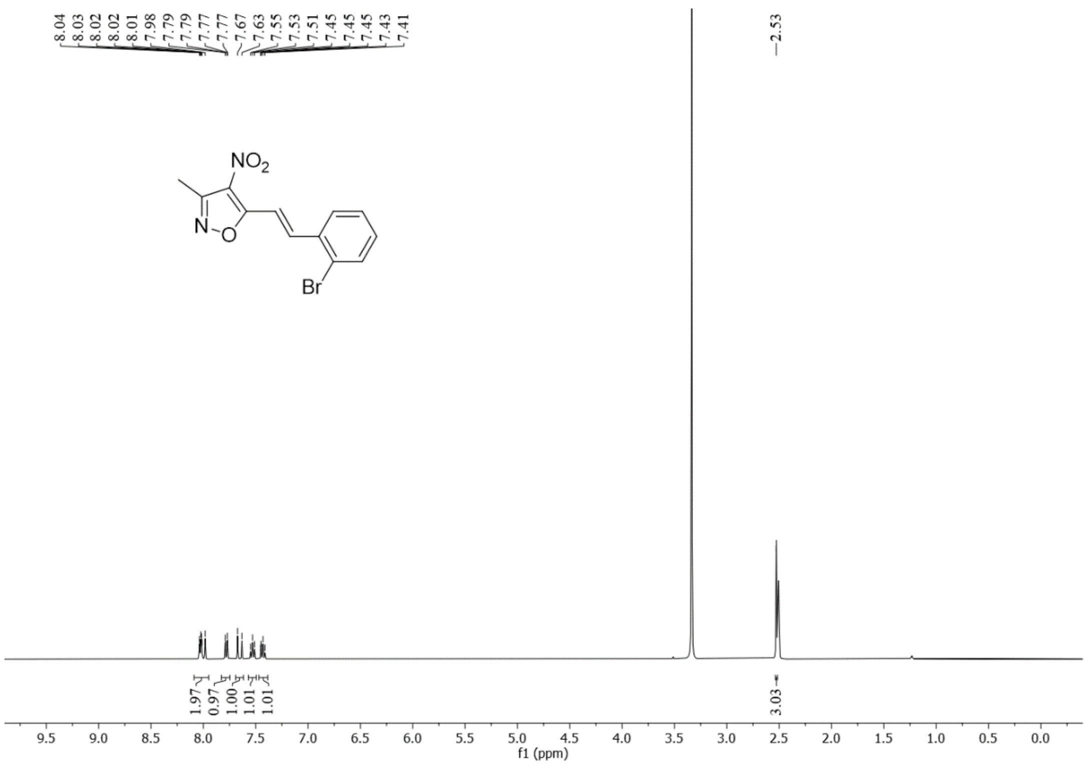

^{13}C-NMR spectrum of **15**

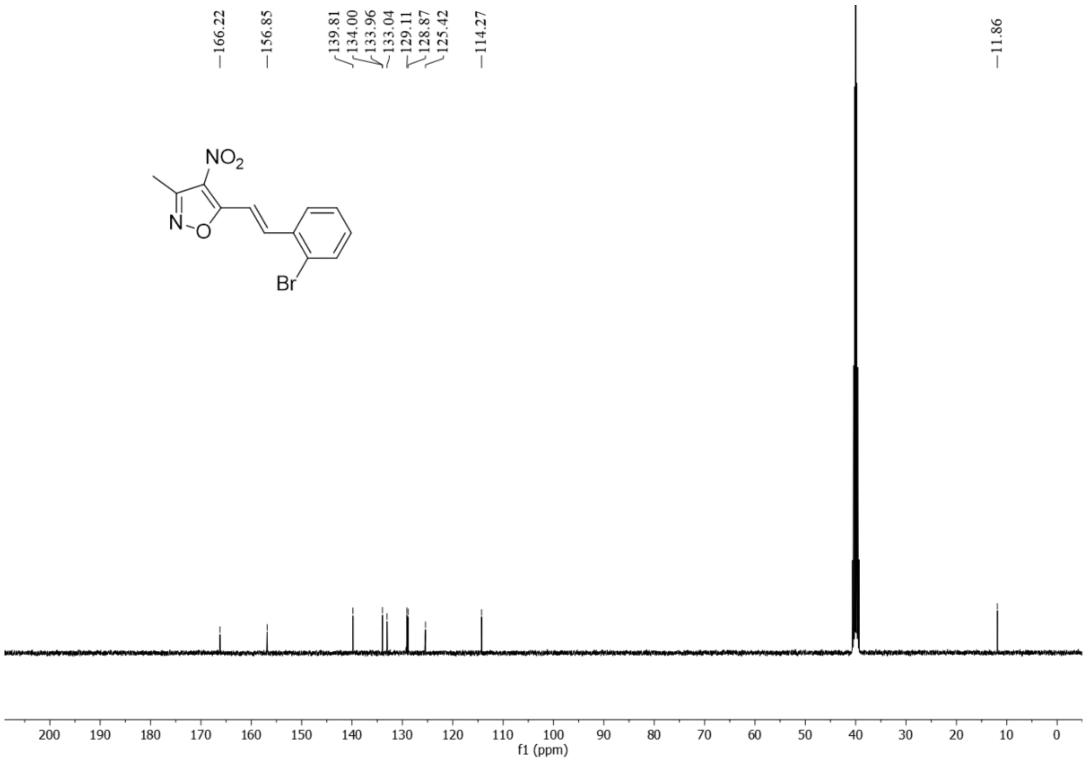

^{1}H-NMR spectrum of **16**

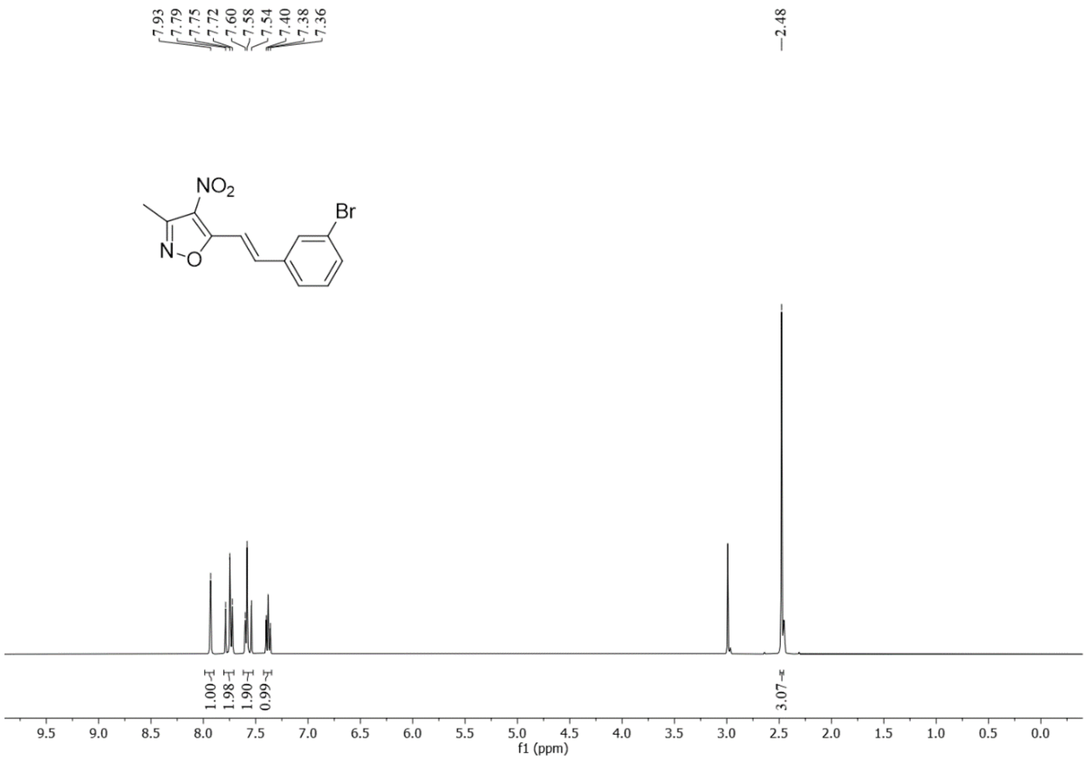

^{13}C-NMR spectrum of **16**

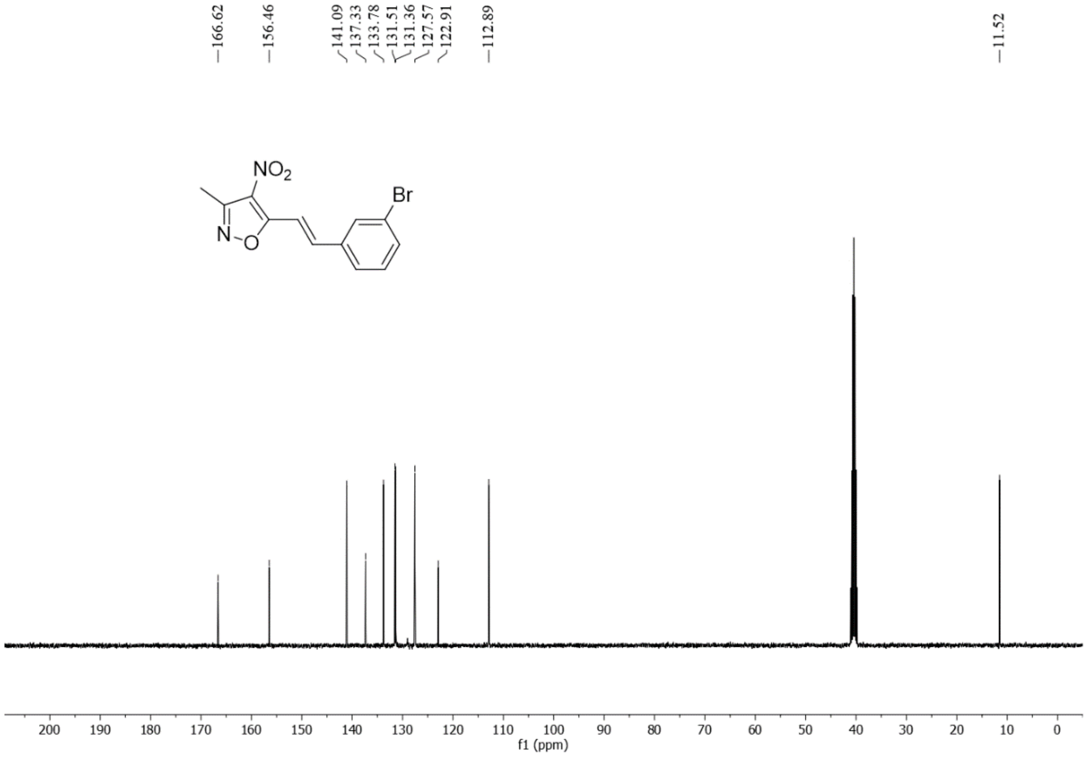

^{1}H-NMR spectrum of **17**

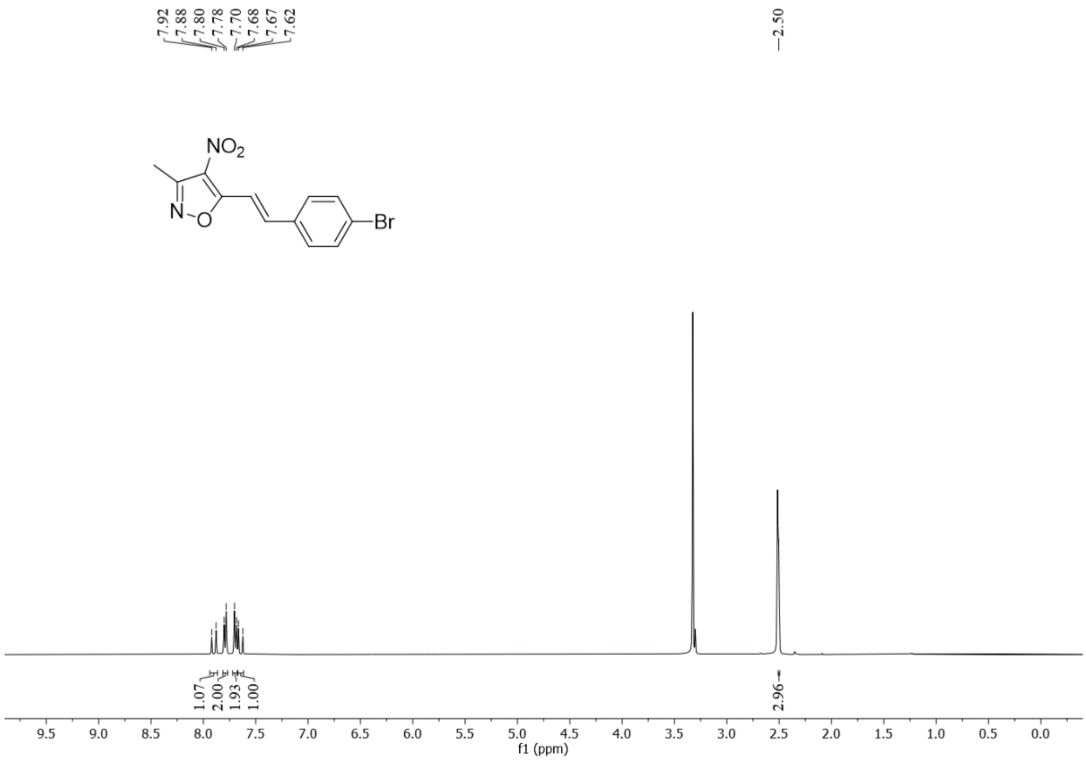

^{13}C-NMR spectrum of **17**

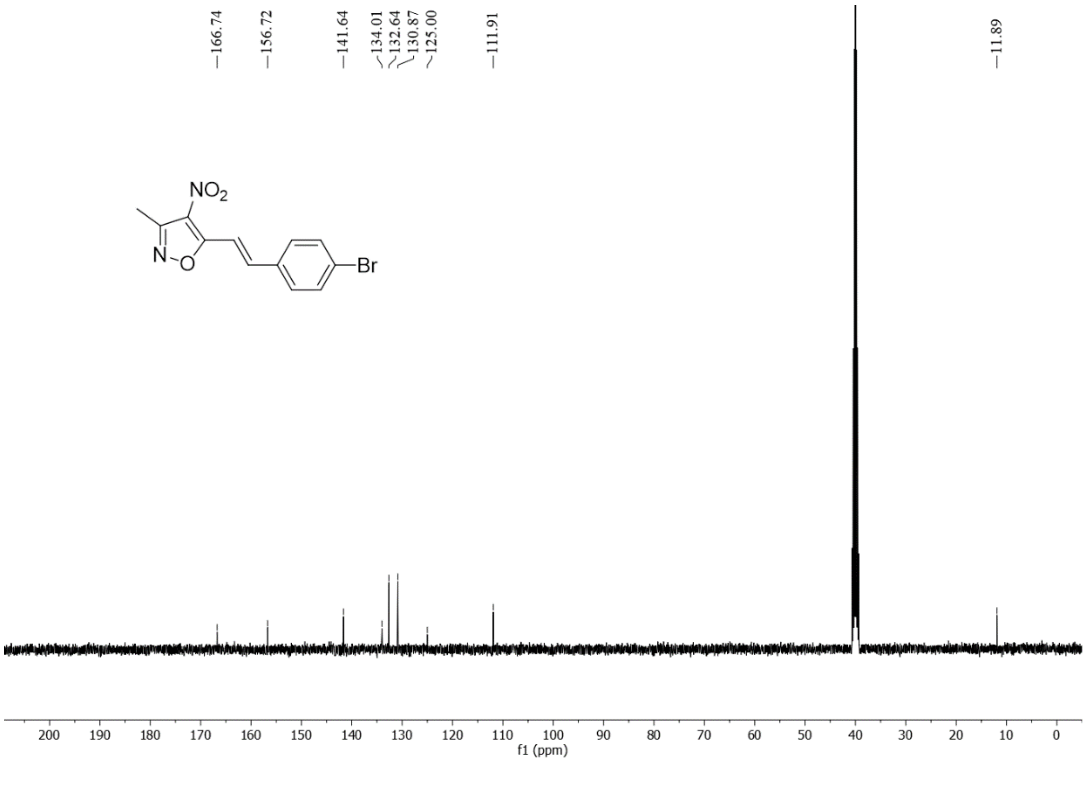

Figure S2: Profile Physicochemical properties

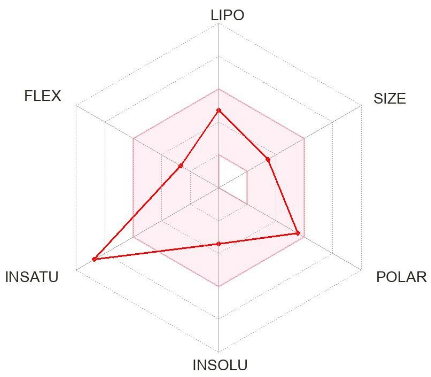

Representative profile of physicochemical properties of the Nitroisoxazole series, the delimited area represents the optimal range for each property (the limits of each property are: Lipophilicity (expressed from the XLOGP3 variable) between -0.7 and +0.5, Size: between 150 and 500g/mol, polarity (TPSA) between 10 to 130 A°2, Solubility (Log S) no greater than 6, unsaturations (fraction of carbons with SP3 hybridization) no greater than 0.25, Flexibility no more than 9 rotatable bonds .

Molecule 1

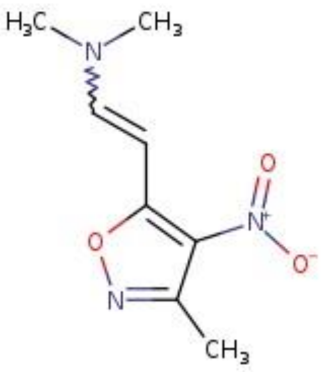

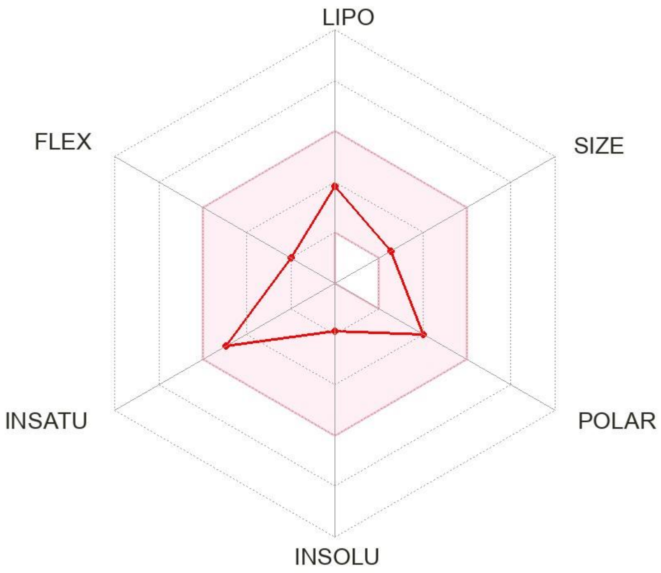

Molecule 2

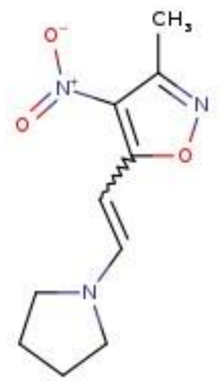

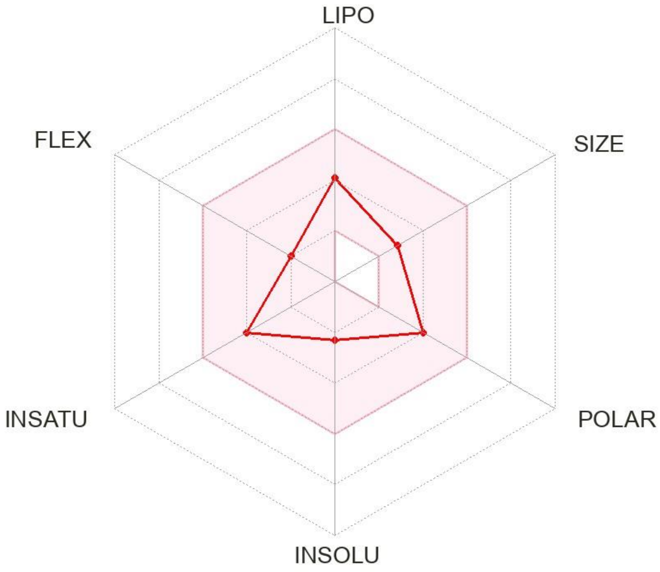

Molecule 3

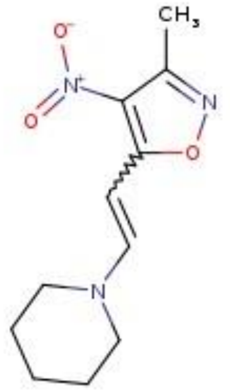

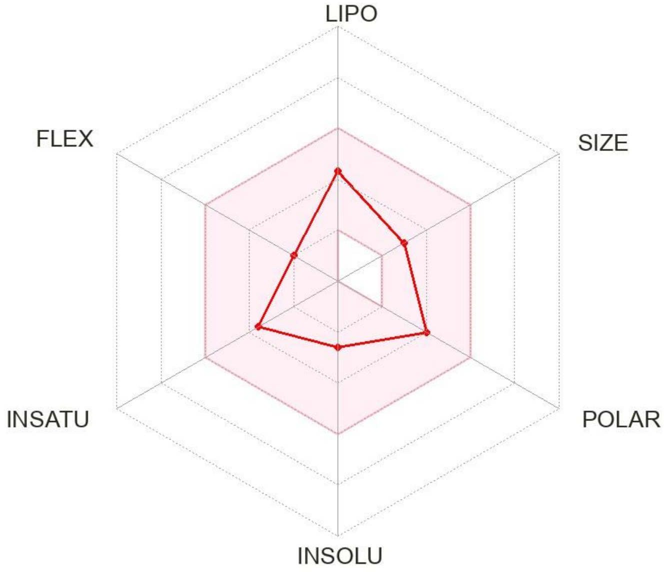

Molecule 4

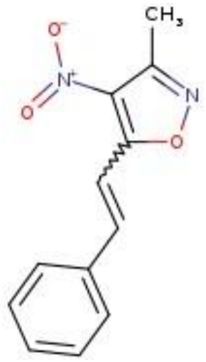

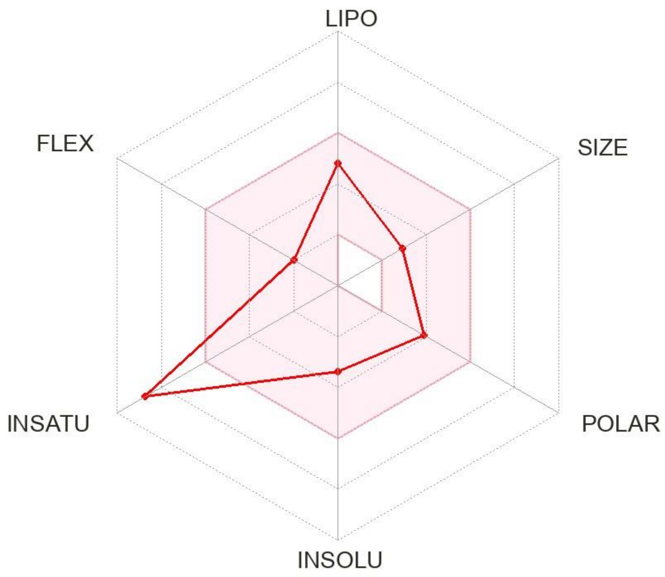

Molecule 5

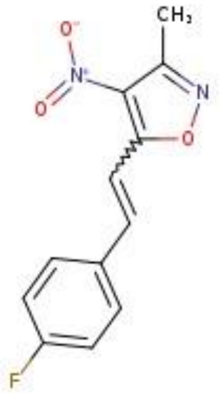

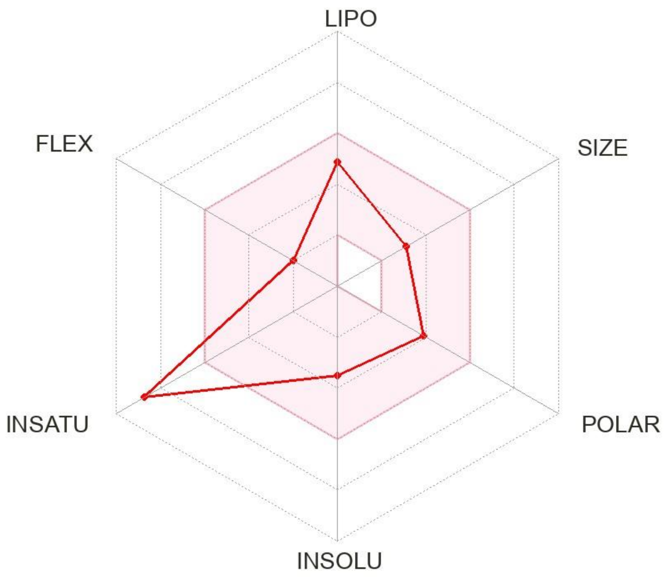

Molecule 6

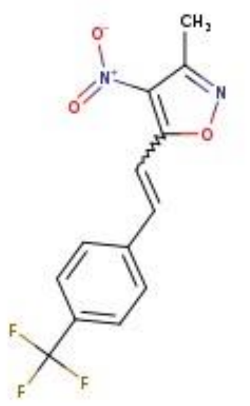

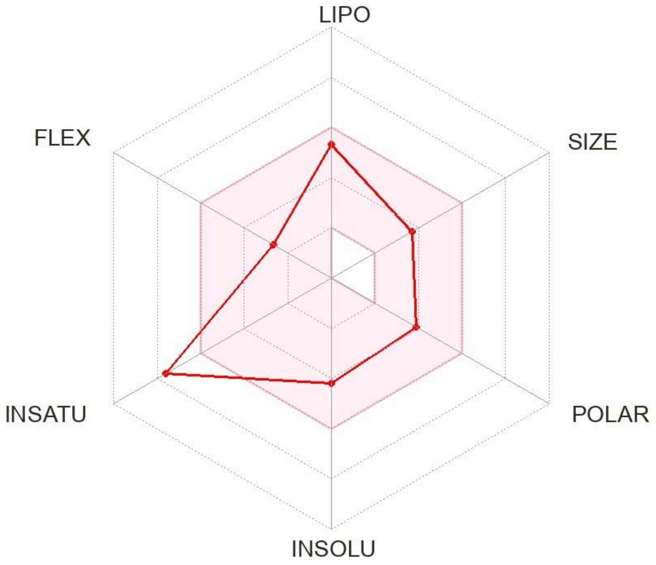

Molecule 7

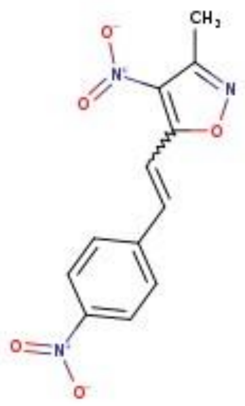

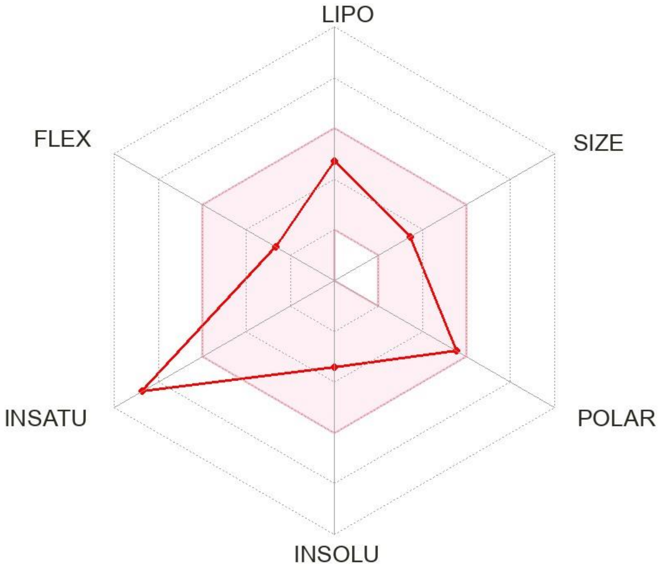

Molecule 8

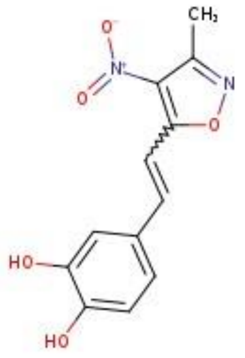

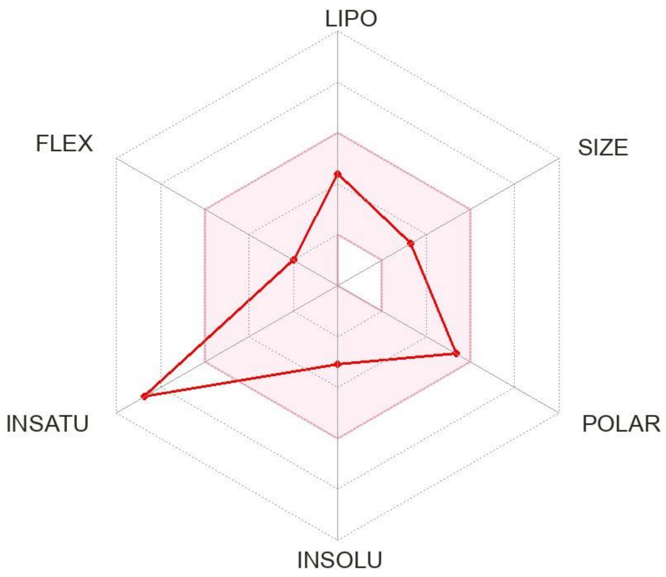

Molecule 9

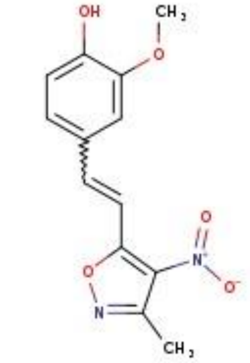

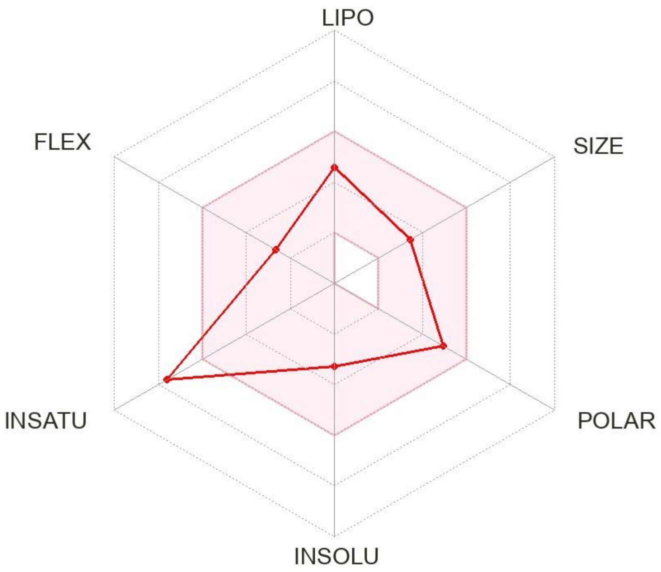

Molecule 10

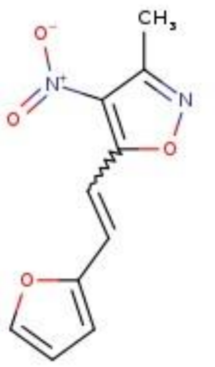

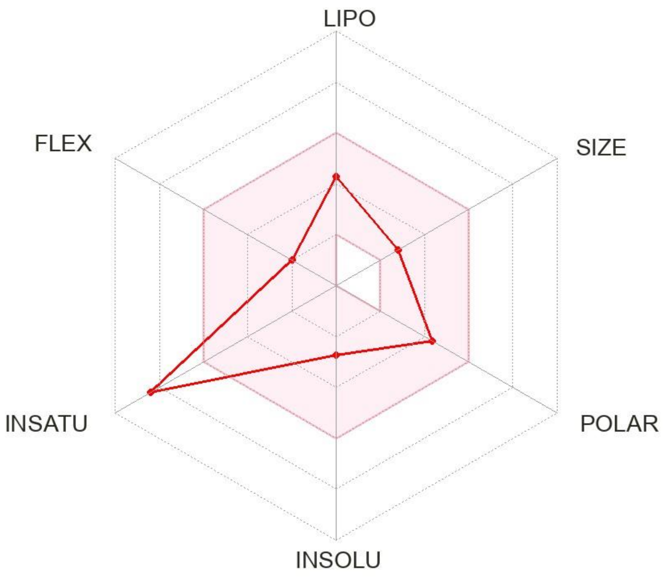

Molecule 11

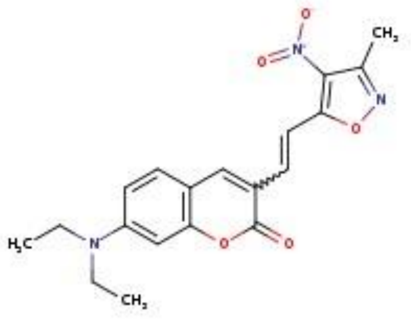

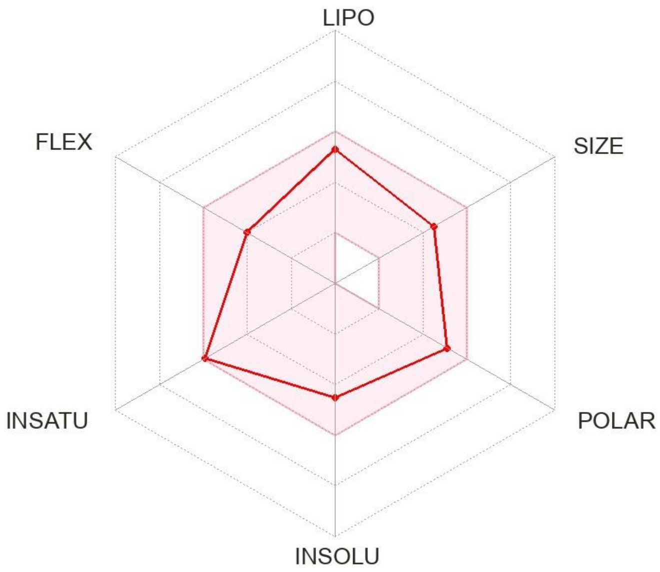

Molecule 12

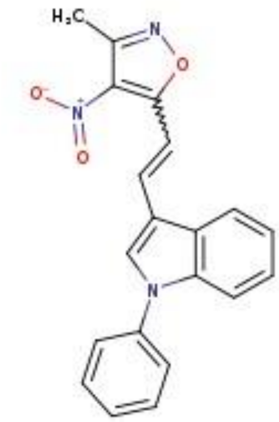

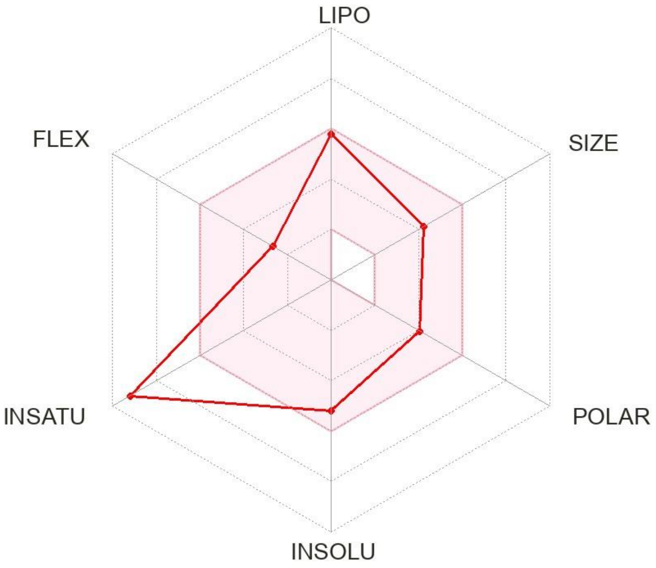

Molecule 13

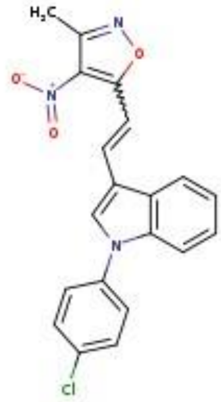

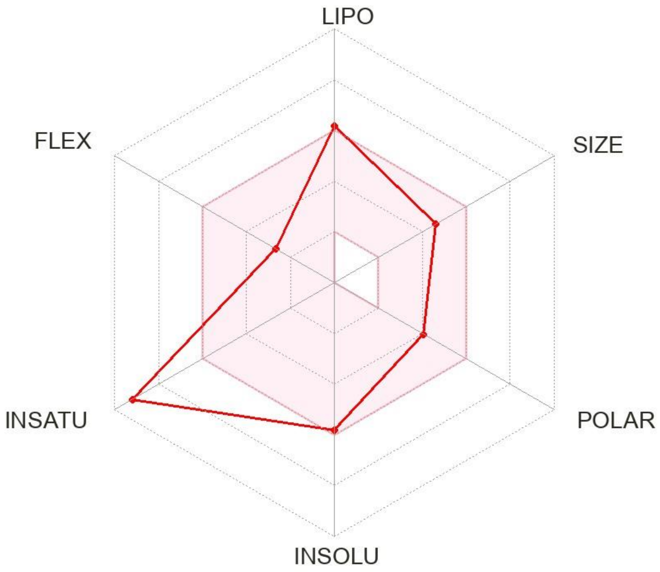

Molecule 14

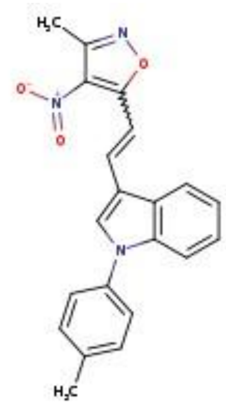

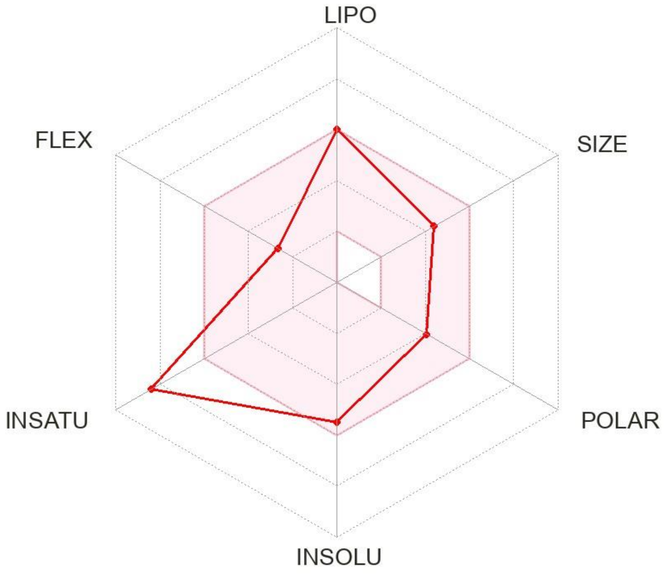

Molecule 15

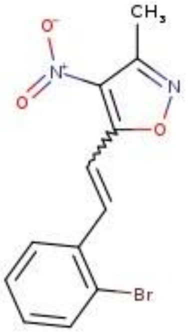

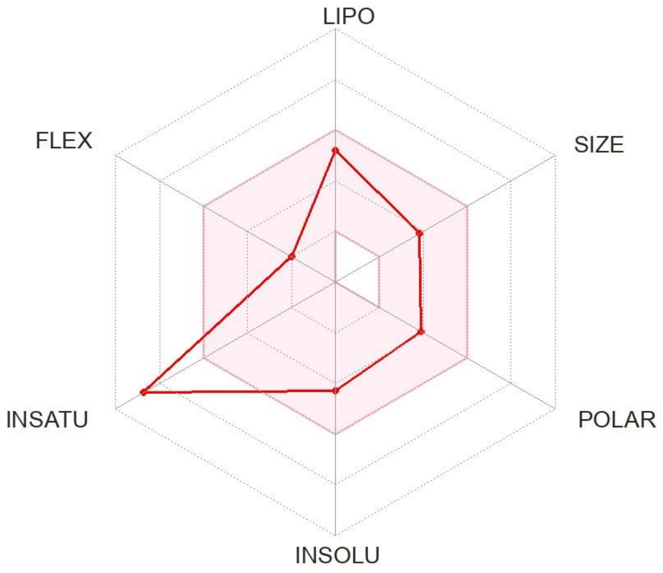

Molecule 16

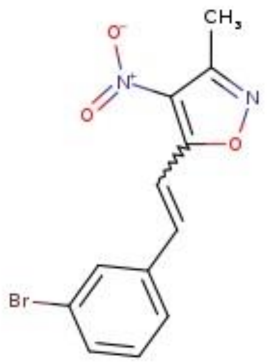

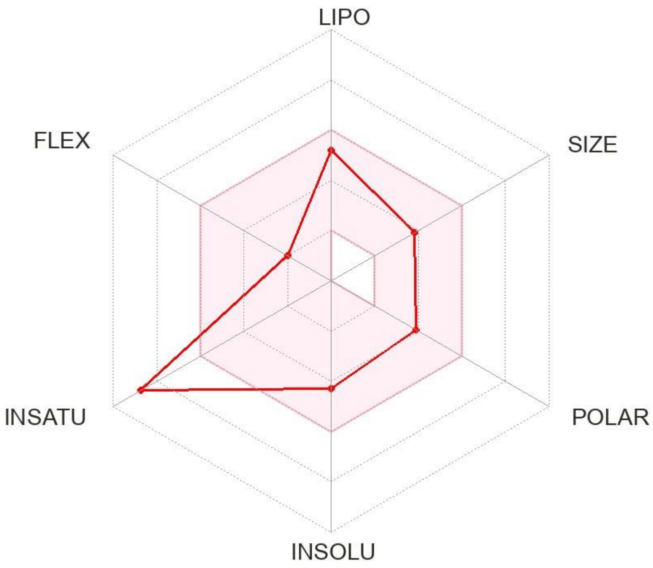

Molecule 17

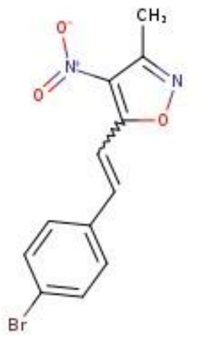

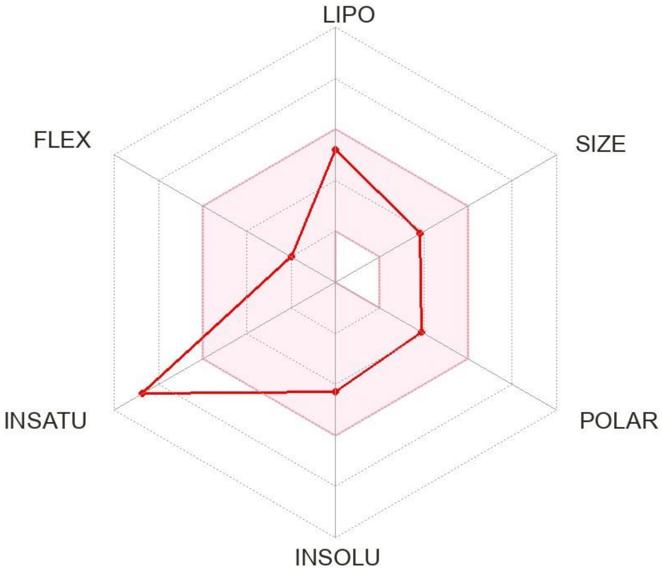

Supplement: Supplementary file 1 [file molecules-29-02762-s001.zip › molecules-3028202-supplementary.pdf]
